# The characteristics of insoluble softwood substrates affect fungal morphology, secretome composition, and hydrolytic efficiency of enzymes produced by *Trichoderma reesei*

**DOI:** 10.1186/s13068-021-01955-5

**Published:** 2021-04-26

**Authors:** Vera Novy, Fredrik Nielsen, Daniel Cullen, Grzegorz Sabat, Carl J. Houtman, Christopher G. Hunt

**Affiliations:** 1 US Department of Agriculture, Forest Products Laboratory, One Gifford Pinchot Drive, Madison, WI 53726 USA; 2 University of Wisconsin Biotechnology Center, Madison, WI 53706 USA; 3 Present Address: Department of Biology and Bioengineering, Division of Industrial Biotechnology, Chalmers University of Technology, Kemivägen 10, 412 96 Göteborg, Sweden

**Keywords:** Softwood substrates, Enzyme production, *Trichoderma reesei*, Secretome, Substrate sensing, Enzymatic hydrolysis

## Abstract

**Background:**

On-site enzyme production using *Trichoderma reesei* can improve yields and lower the overall cost of lignocellulose saccharification by exploiting the fungal gene regulatory mechanism that enables it to continuously adapt enzyme secretion to the substrate used for cultivation. To harness this, the interrelation between substrate characteristics and fungal response must be understood. However, fungal morphology or gene expression studies often lack structural and chemical substrate characterization. Here, *T. reesei* QM6a was cultivated on three softwood substrates: northern bleached softwood Kraft pulp (NBSK) and lodgepole pine pretreated either by dilute-acid-catalyzed steam pretreatment (LP-STEX) or mild alkaline oxidation (LP-ALKOX). With different pretreatments of similar starting materials, we presented the fungus with systematically modified substrates. This allowed the elucidation of substrate-induced changes in the fungal response and the testing of the secreted enzymes’ hydrolytic strength towards the same substrates.

**Results:**

Enzyme activity time courses correlated with hemicellulose content and cellulose accessibility. Specifically, increased amounts of side-chain-cleaving hemicellulolytic enzymes in the protein produced on the complex substrates (LP-STEX; LP-ALKOX) was observed by secretome analysis. Confocal laser scanning micrographs showed that fungal micromorphology responded to changes in cellulose accessibility and initial culture viscosity. The latter was caused by surface charge and fiber dimensions, and likely restricted mass transfer, resulting in morphologies of fungi in stress. Supplementing a basic cellulolytic enzyme mixture with concentrated *T. reesei* supernatant improved saccharification efficiencies of the three substrates, where cellulose, xylan, and mannan conversion was increased by up to 27, 45, and 2800%, respectively. The improvement was most pronounced for proteins produced on LP-STEX and LP-ALKOX on those same substrates, and in the best case, efficiencies reached those of a state-of-the-art commercial enzyme preparation.

**Conclusion:**

Cultivation of *T. reesei* on LP-STEX and LP-ALKOX produced a protein mixture that increased the hydrolytic strength of a basic cellulase mixture to state-of-the-art performance on softwood substrates. This suggests that the fungal adaptation mechanism can be exploited to achieve enhanced performance in enzymatic hydrolysis without a priori knowledge of specific substrate requirements.

**Supplementary Information:**

The online version contains supplementary material available at 10.1186/s13068-021-01955-5.

## Background

Lignocellulose, due to its renewability, abundance, and low cost, is the feedstock choice for more sustainable production of chemicals and fuels [1, 2]. A major economic challenge for commercial biorefinery processes is the high cost of saccharification. This cost driver applies to both lignocellulosic feedstock in general and to more recalcitrant softwood feedstocks, in particular, which require more energy and chemically intense pretreatments and higher enzyme loadings to achieve high sugar yields [3]. Lowering protein production costs and increasing cellulolytic efficiency of enzyme mixtures are two ways to lower saccharification costs [4–7]. We have recently argued that co-location of enzyme manufacturing with biorefineries and integration of intermediate material process streams between them can address both issues [8]. The immediate economic advantages include reduced supplier dependencies and reduced costs for enzyme mixture formulation, transportation, and storage [7, 9, 10]. Furthermore, using the biorefinery substrate as carbon source for enzyme production, the microorganism’s natural mechanisms of adaptation can be exploited to produce substrate-specific and more efficient enzyme mixtures [8].

Through its complex gene regulation machinery, *Trichoderma reesei*, the principal fungus exploited to produce commercial enzymes [11], adjusts the composition of secreted enzymes to match the substrate characteristics [12–20]. The fungus depends on metabolizable mono- and disaccharides released by enzymatic feedstock hydrolysis for carbon and energy. These small molecules also act as inducers for gene expressions [8, 21–23]. Because feedstock characteristics vary drastically and change continuously, the gene regulatory machinery is essential for the fungus to adapt and finetune its enzyme expression and secretion pattern to live off lignocellulose. However, to harness the adaptation potential of *T. reesei*, a more in-depth understanding of the interrelation between feedstocks’ structural and chemical properties and fungal gene regulatory mechanisms is required [8, 21, 24].

Previous research suggests, often indirectly [8], that chemical composition [25–27] and substrate ultrastructure [18] affect enzyme titers and productivities. It has been shown, using advanced “omics” techniques, that (hemi-)cellulose derived molecules trigger specific gene regulatory responses in *T. reesei*, resulting in changes in the transcriptome and secretome [8, 21, 23, 24, 28–30]. Similar to other filamentous fungi [31, 32], protein productivity and fungal growth in *T. reesei* are closely connected to the fungal morphology and the “environome” (i.e., the conditions under which the fungus is cultivated). Thus, the impacts of media composition [13, 33, 34], pH [35], agitation [36, 37], and light [24] on fungal growth and protein production have been identified. Differences in fungal morphology, typically divided into macro-scale (i.e., dispersed or pelletized growth) and micro-scale (i.e., single cell dimensions and ramification) occurrences [32, 38], have been analyzed and related to variations in the environome [18, 33, 39, 40]. Quantitative metrics of these morphological properties have been used to establish correlations to enzyme productivity in *T. reesei* cultivation [33, 36, 41].

To derive a comprehensive view of effects induced by the environome from research conducted on lignocellulosic substrates [30, 42–44], we recently systematically collected and appraised data on enzyme production by *T. reesei* [8]. Although protein production and single enzyme activities could be correlated to inferred substrate characteristics [8], detailed analyses were limited by the lack of information on the substrate’s chemical and structural properties [8]. In this study, we provide an improved understanding of the effects of the substrates’ chemical composition and structural properties on fungal morphology, enzyme productivity, and enzyme secretion profiles in cultivations of *T. reesei*. Furthermore, we investigate the hydrolytic performance of the produced protein on the same substrates to assess the general and substrate-specific softwood hydrolysis efficiency. Specifically, we focused on softwood materials, for which the need for improved cellulolytic efficiency is most pressing. Three pretreated materials were characterized and used: commercial northern bleached softwood kraft pulp (NBSK), dilute-acid-catalyzed steam-pretreated Lodgepole pine (LP-STEX), and mild alkali-oxygen-treated Lodgepole pine (LP-ALKOX). While starting with similar wood, the three pretreatments provide different fiber size distributions, morphologies, surface charges, and chemical compositions.

## Results and discussion

### Pretreatment and substrate characteristics

**Fig. 1:**
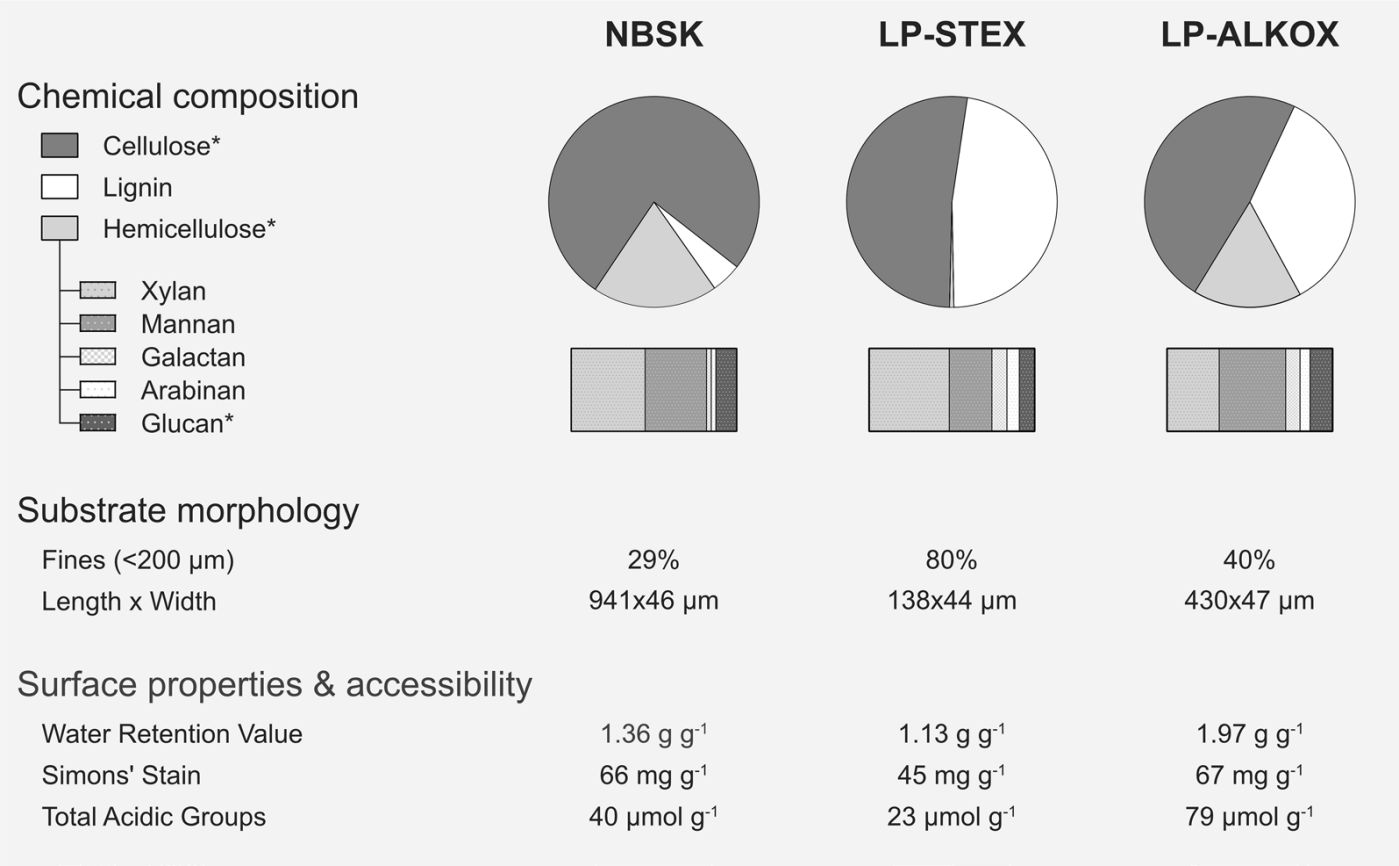
Summary of properties of the softwood substrates. The chemical compositions are given as % of dry weight. The glucan fraction of hemicellulose was estimated based on the mannopyranose-to-glucopyranose ratio in softwood galactoglucomannan [45], to account for glucose that was released from galactoglucomannan rather than cellulose. The cellulose and hemicellulose items were adjusted accordingly (*). Additional substrate characteristics were measured to profile the substrates’ fiber swelling (water retention value), accessibility (Simons’ stain), and surface charge (total acidic groups). Detailed information is given in Additional file 1: Table S1, Table S2, and Figure S1

The chemical compositions and physical structures of the three substrates governed hydrolyzability, cultivation conditions, and availability of carbon source to the fungus. The substrate preparation methods produced different chemical compositions and spatial distributions, as well as different substrate morphologies, while other factors, e.g., lignin S/G ratio, were constant because of the similar starting materials. This allowed a clearer evaluation of the impacts of defined parameters. The substrates’ properties are summarized in Fig. 1 and detailed in Additional file 1: Table S1 and S2.

NBSK was enriched in cellulose (75 wt%) and with partially retained hemicellulose (19 wt%) and lignin (≤ 4.6 wt.%). The fibers contain mostly ordered crystalline regions with less ordered cellulose on surfaces [46], interspersed with mechanically damaged, less ordered zones [47]. The retained hemicellulose largely comprised the more recalcitrant xylan and glucomannan backbones (Fig. 1). The lignin component was removed by pulping and bleaching processes. These processes can result in some reprecipitated lignin and extractives on fiber surfaces [46]. The measured residual lignin content was likely overestimated by interference from chromophoric carbohydrate dehydration products in determination of acid-soluble lignin [48], which comprise ≤ 98% of measured lignin (Additional file 1: Table S1). The fiber morphology was characterized by longer fibers ($\overline{x }$= 941 µm, Additional file 1: Figure S1) and the widths were largely determined by the width of the swollen tracheid ($\overline{x }$= 46 µm). The total acid group content of NBSK (40 µmol g^−1^) mainly originates in residual hexenuronic acid groups on xylan [49] and, to lesser extent, carboxylic groups introduced in lignin and (hemi-)cellulosic polymers during oxygen-bleaching stages [50].

LP-STEX was enriched in cellulose (52 wt.%) and lignin (47 wt.%). Hydrolysis of glycosidic bonds in hemicellulosic polymers during pretreatment caused almost complete dissolution of hemicellulose, which comprise < 0.7 wt.% of the resulting substrate (Table S1). The cellulose component was affected to a much lesser extent. In the process, groups on hemicellulose and lignin were catalytically cleaved to various extent [51]. The lignin component was partially solubilized and a condensed lignin, typically hydrophobic [52] and less prone to acidolysis, formed by cycles of de- and repolymerization reactions [53]. On a structural level, pretreatment disrupted the cell wall structure, melted and redistributed lignin onto surfaces, and caused fragmentation of the pretreated material [52, 54, 55]. The resulting fiber morphology was heterogeneous with small particles ($\overline{x }$=138 µm, Additional file 1: Figure S1), comprised 80% fines, and contained some non-defibrated wood chip fragments. The LP-STEX had low amounts of total acid groups (23 µmol g^−1^).

LP-ALKOX retained cellulose, hemicellulose, and lignin components in the fibers (Additional file 1: Fig. 1; Table S1). The minor changes in chemical composition were due to removal of labile extractives and minor solubilization of hemicellulose and lignin components (Additional file 1: Table S1). Chemical modification of lignin and mechanical refining were used to improve susceptibility to enzymatic deconstruction. The proposed effect of alkali-oxygen treatment is incorporation of carboxylic acid end-groups in the lignin macromolecule by fragmentation-, side-chain eliminating-, and ring-opening reactions between lignin and oxygen species, analogous with oxygen bleaching [50]. Mechanical pulping broke up the wood matrix and fibrillated fibers, which increased the effective surface of the substrate. The LP-ALKOX comprised 40% fines and a morphology characterized by longer fibers ($\overline{x }$=430 µm, Additional file 1: Figure S1) and similar fiber width ($\overline{x }$=47 µm) as NBSK and LP-STEX (Fig. 1). The high content of acidic groups (79 µmol g^−1^) has its origins in incorporation of carboxylic groups in the lignin macromolecule and (hemi-)cellulosic polymers [50] and likely in uronic acid residues on xylan, analogous with alkaline cooking [49].

Differentially retained hemicellulose and lignin and altered physical structures of the biomass created an array of substrates with increasing complexity, from NBSK over LP-STEX to LP-ALKOX. The alterations affect accessible interior and exterior surface areas of exposed cellulose, assayed by Simons’ staining (Fig. 1), and thus, the hydrolyzability of substrates and rate of hydrolysis. Smaller particle sizes increase exterior surface areas and increase accessibility to enzymes [56], affecting the substrates to various extent (LP-STEX > LP-ALKOX > NBSK). Furthermore, lignin and hemicellulose act as physical barriers that limit cellulose accessibility [56], and their removal and redistribution in the wood matrix can decrease accessibility constraints. NBSK relies on delignification to expose the cellulose component. Meanwhile, removal of hemicellulose and redistribution of lignin in LP-STEX increase accessibility of the fiber bulk [56]. Simultaneously, redeposition of lignin onto exterior surfaces masks the fiber and decreases accessibility in the initial stages of hydrolysis [57]. LP-ALKOX maintains components and structural complexity and relies on lignin rearrangement and increased interior porosity from charged groups. Bulk charges induce fiber swelling by electrostatic repulsions [58] and have been shown to be important for accessibility of cellulose to enzymes [56]. The water retention value provides a proxy for total fiber swelling (Fig. 1). Furthermore, exterior surface charges affect inter-fiber interactions and consequently fiber flocculation and rheology in cultivations and enzymatic hydrolyzes [59]. The characterization delineates the substrates’ properties at onset of fungal cultivation and enzymatic hydrolysis. However, as enzymatic hydrolysis progresses and various activities exert their actions, these properties will continuously change. Thus, the temporal dimension of the substrates’ chemical composition and physical structure have effects on cultivations and hydrolytic efficiency.

### The physical interaction between *T. reesei* and insoluble substrates

The fungal cultivations were performed in bioreactors to minimize mass and heat transfer constraints, which can negatively affect enzyme productivities and titers [8]. The cultivation supernatants were characterized with respect to total proteins, enzymatic activities, final secretome composition, and hydrolytic efficiency on the softwood substrates. Furthermore, changes in fungal micromorphology in response to the softwood substrates and the physical interaction between fungal hyphae and the insoluble substrates were investigated with CLSM.

### The impact of softwood substrates on fungal micromorphology

**Fig. 2:**
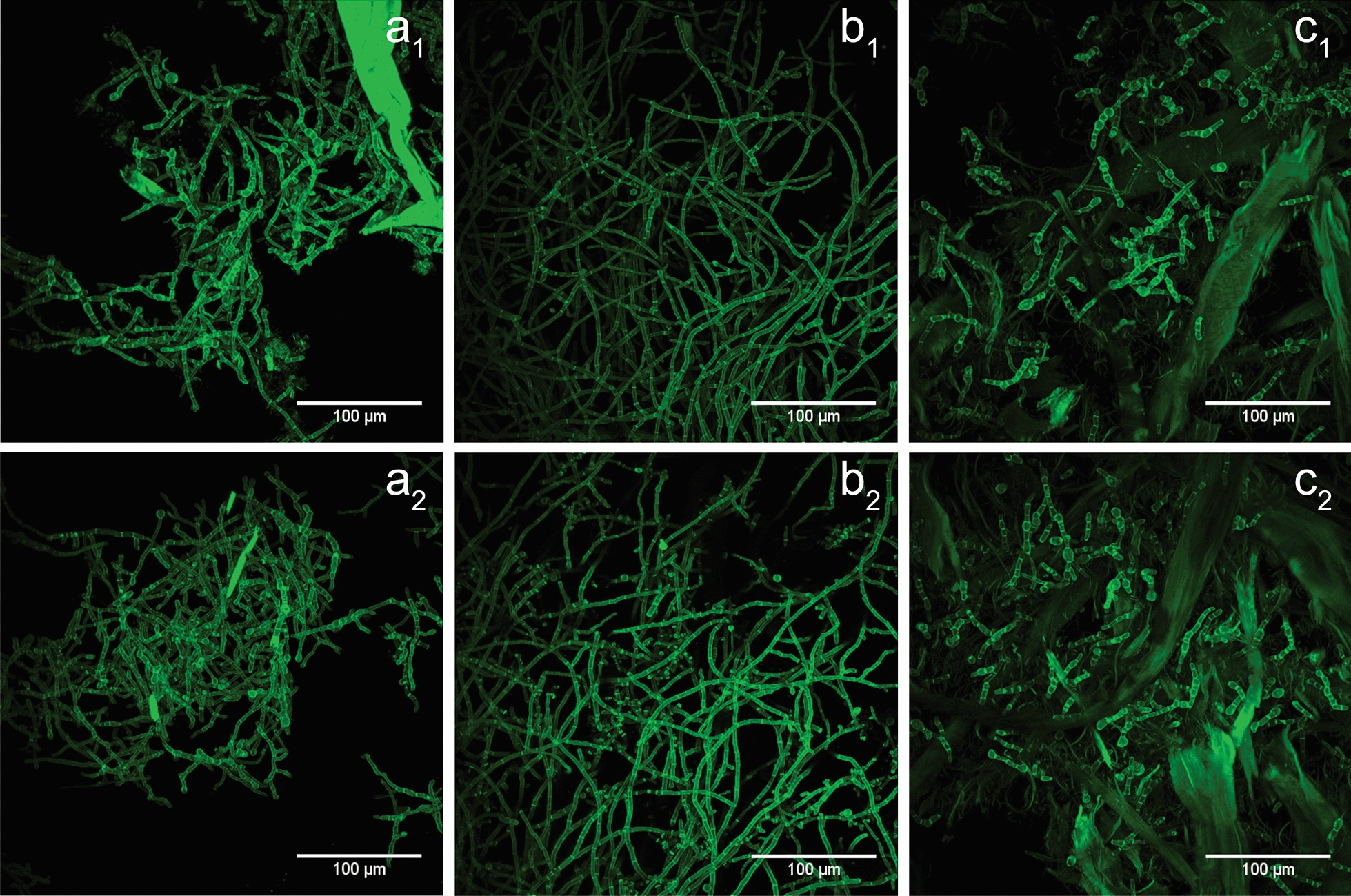
CLSM micrographs of fungal morphology after 48 h (**a**_1_, **b**_1_, **c**_1_) and 96 h (**a**_2_, **b**_2_, **c**_2_) in *T. reesei* QM6a cultivation on NBSK (a_1_, a_2_), LP-STEX (**b**_1_, **b**_2_), and LP-ALKOX (**c**_1_, **c**_2_). Staining was performed with Calcofluor white stain (CF). Colors are assigned arbitrarily

Fungal morphology has been connected to fungal growth and protein productivity [32, 38]. On a macroscopic scale, the fungus can grow dispersed or pelletized and, on a microscopic level, morphology can be assessed by parameters such as the single cell size (length, width, and volume) and the degree of ramification [32, 38]. The cultivations exhibited dispersed growth, likely because the insoluble substrates prevented the aggregation of spores and hyphae required for pelletized growth [60, 61]. Differences in micromorphological development of the fungal hyphae induced by characteristics of the insoluble substrates were investigated by CLSM imaging of CF stained hyphae (Fig. 2).

**Fig. 3:**
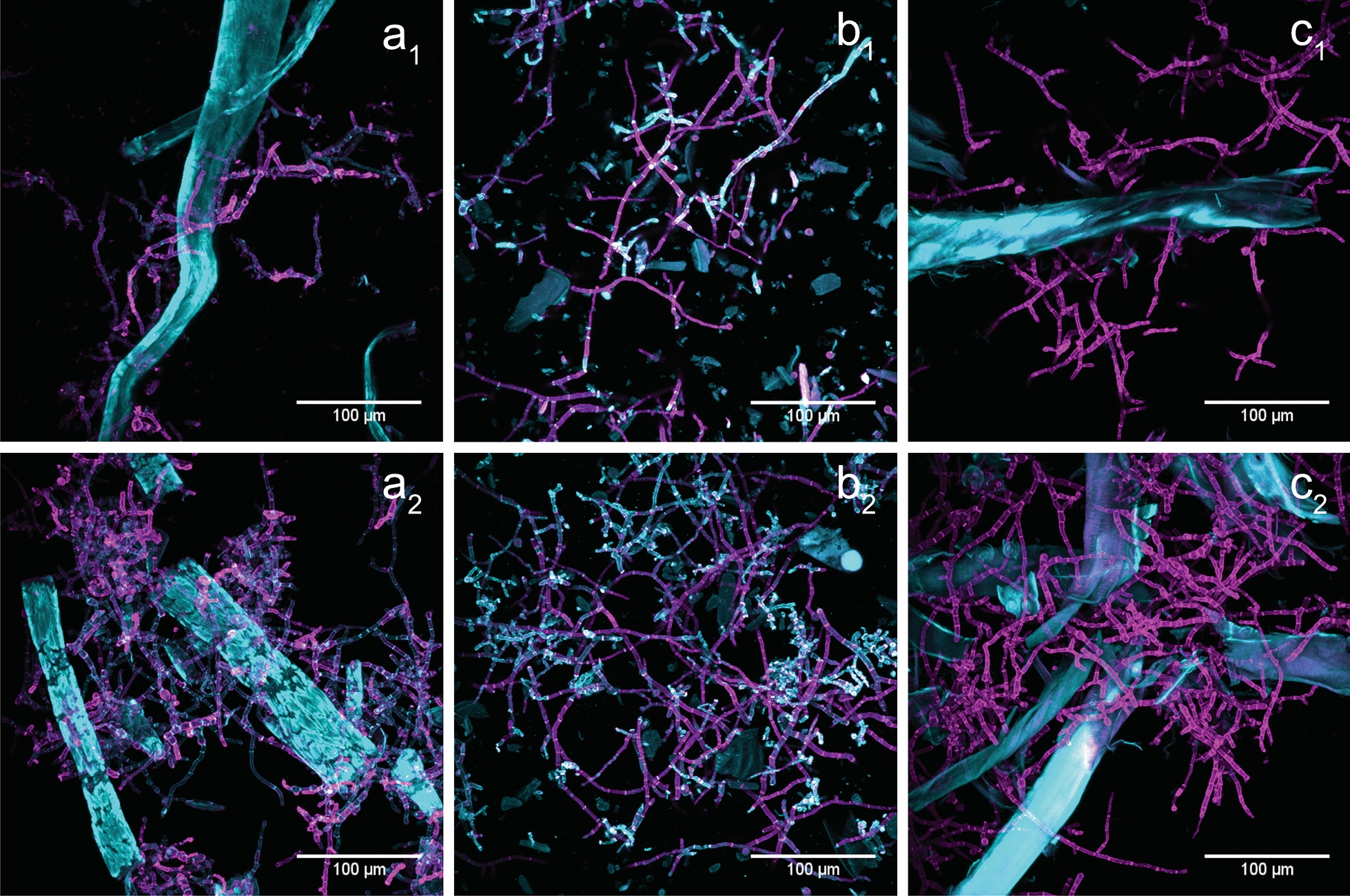
CLSM micrographs of hyphae–substrate interaction in cultivations of differentially stained *T. reesei* QM6a on NBSK (**a**_1_, **a**_2_), LP-STEX (**b**_1_, **b**_2_), and LP-ALKOX (**c**_1_, **c**_2_). Fungal hyphae are depicted in magenta, and the substrate solids in turquois. Colors are assigned arbitrarily. Samples were taken after 48 h (**a**_1_, **b**_1_, **c**_1_) and 72 h (**a**_2_) and 96 h (**b**_2_, **c**_2_)

On LP-ALKOX and NBSK, but not on LP-STEX, the fungus developed bulbous cells, which have been described previously [18, 35] and were suggested to be caused by formation of a thick fibrous outer cell wall layer [62]. This morphology has been related to a variety of factors, such as lack of nutrients, starvation, and stress [18, 31, 35]. Furthermore, it has been suggested that the presence of lignocellulose results in formation of thick cell walls in *T. reesei* to be able to anchor more enzymes in the outer cell wall [63] and, thus, increase the cellulolytic capacity of the fungus [62]. Although the underlying reason warrants further investigation, the observed bulbous cell growth was likely induced by nutritional stress. The flocculation of NBSK fibers [64] elicited a shear-thinning and viscous medium (Additional file 1: Figure S2) and interactions with the hyphal network added to these properties [65]. The rheology of the media likely gave rise to heterogeneous, non-mixed zone formation, thus limiting mass transfer of oxygen and nutrients available to the cells. This notion is supported by a previous study on pulp [18] and the observed decrease in relative number of bulbous cells over time (*cf*. Figure 2 a1 and a2), where onset of enzyme-mediated fiber fragmentation (*cf*. Figure 3 a1 and a2) reduced the media viscosity significantly. Bulbous cell growth was induced on LP-ALKOX by high viscosity (Additional file 1: Figure S2), analogous with NBSK. In addition, higher recalcitrance of LP-ALKOX to enzymatic hydrolysis, as will be shown below, resulted in delayed fiber fragmentation and slower release of sugars, which also may contribute to nutrient deficiency.

The substrates elicited further micromorphological differences. From LP-STEX over NBSK to LP-ALKOX, the total length of hyphae, degree of ramification, and single cell lengths decreased (Fig. 2). The observed decrease in hyphal length thereby was likely a function of cell growth and fragmentation [60]. The viscous media with NBSK and LP-ALKOX required higher agitation intensities (300–500 rpm) than LP-STEX (200–300 rpm) to provide mixing and meet dissolved oxygen demand, which increased hydrodynamic and mechanical shear forces, which likely led to increased damage to mycelia and fragmentation [32, 36]. Ramification is a micromorphological parameter that is often described to strongly correlate with enzyme production. A higher degree of branching thereby has been suggested to increase protein production, because protein secretion mainly happens at the spitzenkörper, i.e., freshly formed tips [66]. Comparing the three types of fibers, LP-ALKOX had the lowest amount of ramification, and as has been previously observed, LP-STEX and NBSK resulted in higher protein production than LP-ALKOX. Finally, it has been shown more recently that cell length affects protein production in *T. reesei* QM9414, where shorter cells were correlated to higher protein yields [41]. In this study, the formed cells were relatively short and wide on NBSK and LP-ALKOX and longer and thinner on LP-STEX. Thus, it appears that in a complex system affected by the substrates’ accessibility and composition, media viscosity, and mass transfer limitations, it is difficult to dissect the effect of a single parameter on fungal morphology. The broad morphological variation observed, however, supports the importance of taking morphological changes into consideration when investigating fungal cultivations, and future studies will include quantitative in-depth analyses.

### The interaction between substrate solids and fungal hyphae

Apart from changes in micromorphology, Fig. 2 suggests a close interaction between fungal hyphae and insoluble substrates, an effect reported previously [41]. To investigate hypha-fiber interaction, CLSM micrographs of differentially stained fungal hyphae and insoluble substrates were acquired (Fig. 3).

On all three substrates, fungal hyphae appear to grow in association with the softwood solids, accumulating insoluble lignocellulosic substrate at the tips of hyphae (Fig. 3). Thus, hyphae grow in and around cracks and holes in wood surfaces and an increased density of hyphal networks can be found around wood solids. Fungi have been described to be able to grow surface-associated [67–69], mainly through secretion of a polysaccharide-containing matrix [70]. Similar to bacterial and yeast-based biofilms [67], formation of a surface-associated layer has been shown to affect gene regulation, among other parameters [68, 69]. However, so far, reports of surface-associated growth have mainly focused on solid-state cultivations [68, 69], which provides profoundly different conditions than submerged cultures [71]. With regard to its effect on protein productivity and gene regulation [68, 69], studying interactions between solid substrates and *T. reesei* will be the focus of future studies.

### The impact of softwood substrates on enzyme production by *T. reesei*

#### Time course analysis of protein concentration and enzymatic activities

**Fig. 4:**
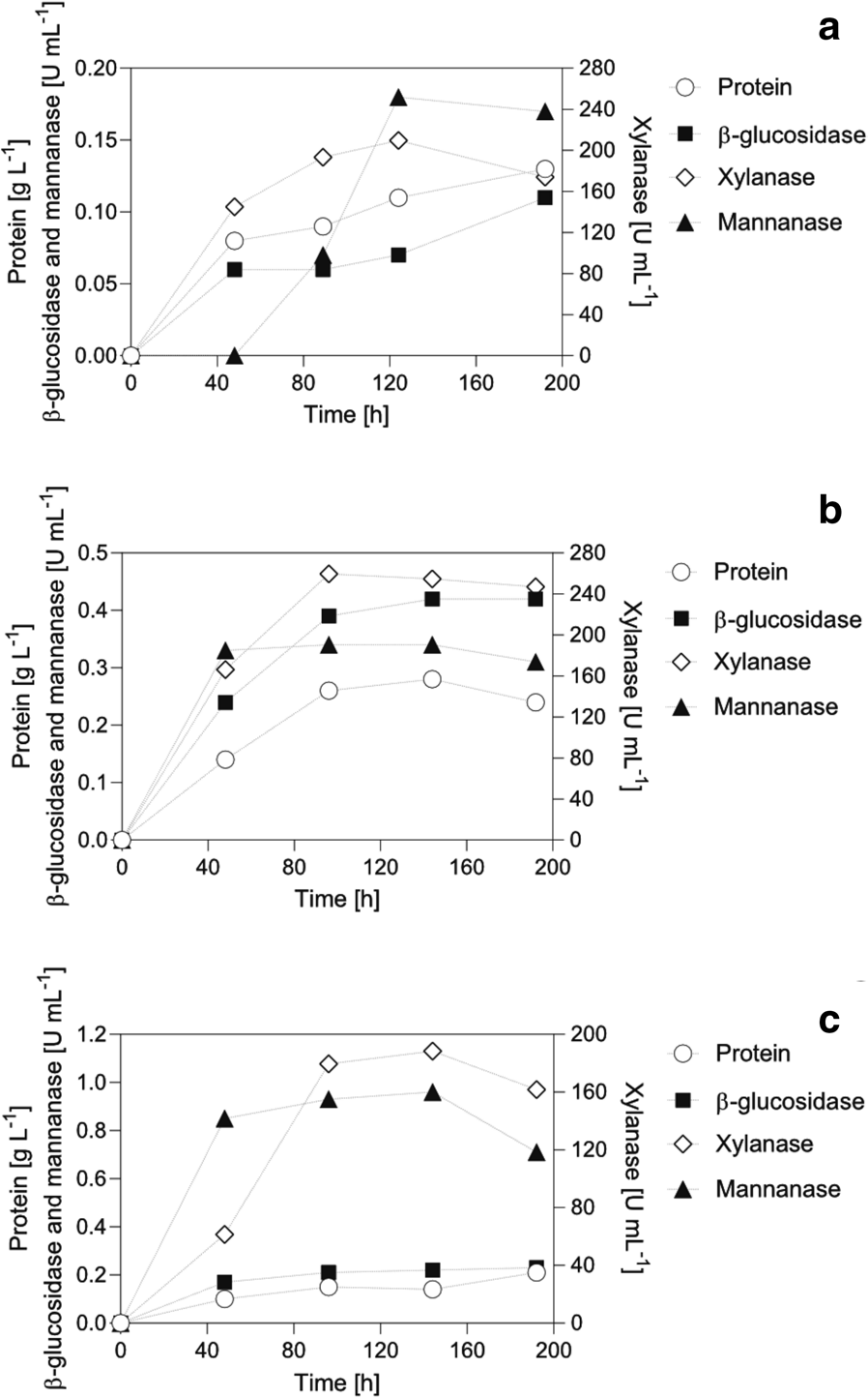
Time courses of protein and activity development in bioreactor cultivations of *T. reesei* QM6a on NBSK (**a**), LP-STEX (**b**), and LP-ALKOX (**c**). Data points are the sum of activities measured in the supernatant and desorbed fraction and represent mean values of technical duplicates. Scales of y-axes have been adjusted for clarity

Time courses of protein concentration and enzymatic activities (comprising supernatant and proteins recovered from substrate solids) are depicted in Fig. 4. When cultivated on lignocellulose, *T. reesei* has been described to show a delayed onset of protein production and biomass growth, an effect ascribed to the gene regulation machinery consisting of sensing, signaling, gene expression, and secretion of (hemi-)cellulolytic enzymes [8, 21, 24, 72]. To avoid the lag phase, we used lactose as carbon source in the precultures. Lactose induces gene expression of a broad set of (hemi-)cellulolytic enzymes [43] because of its similarity to hydrolyzed β-galactoside side chains of xyloglucans [73]. As a result, expressions of (hemi-)cellulolytic enzymes were already induced and metabolizable sugars were released from the softwood from the beginning, which triggered further fungal gene regulatory responses. After an initial increase, protein production plateaued after 48 h (NBSK, Fig. 4a) and 96 h (LP-STEX and LP-ALKOX, Fig. 4b, c). On NBSK, protein production increased again towards the end of cultivation. The ß-glucosidase activity followed the same trend, reaching 0.1, 0.4, and 0.2 U mL^−1^ for NBSK, LP-STEX, and LP-ALKOX, respectively. The xylanase and mannanase secretion were more substrate-dependent, and reached final activities of 210, 255, and 189 U mL^−1^ for xylanases and 0.2, 0.3, and 1.0 U mL^−1^ for mannanase on NBSK, LP-STEX, and LP-ALKOX, respectively.

Based on the substrate characterization, several conclusions can be drawn from the time courses. First, the temporal change in substrate ultrastructure seemed to have affected protein secretion patterns over the course of cultivation. NBSK, which is mainly comprised of cellulose and hemicellulose, initially showed a high cellulose accessibility to enzymes and proteins (Fig. 1) [47]. However, as the cultivation (and hydrolysis) progressed, hemicellulose and disordered cellulose were preferentially removed, as shown in a recent enzymatic hydrolysis study [47], enriching more ordered cellulose with reduced accessibility to enzymes [47]. This led to rate retardation of cellulose degradation as enzymatic hydrolysis became restricted to surface erosion [47, 74]. Thus, we suggest that after an initial burst of high protein productivity, triggered by rapid release of sugars, enrichment of ordered cellulose retarded the sugar release to such an extent that *T. reesei* was forced into starvation at an early stage. This, in turn, resulted in onset of autophagy and release of cell proteins due to a loss in cell wall integrity [41, 43, 75], leading to the observed increase in protein concentration (Fig. 4).

In contrast, redistributed lignin of LP-STEX initially masks fiber surfaces [54] and restricts enzyme access to the cellulose, which is reflected in measured accessibility (Fig. 1). As hydrolysis progressed and porosity increased, the barrier was circumvented. The disruption of plant cell wall structure and dissolution of hemicellulose during pretreatment increased accessibility in subsequent stages and allowed enzymatic hydrolysis to progress via infiltration of the fiber bulk rather than surface erosion [76]. The enzymatic hydrolysis pattern and a high effective surface area of fines resulted in a continuous release of metabolizable sugars, which, in combination with lower viscosity, yielded higher enzyme titers.

LP-ALKOX represented the most complex substrate for *T. reesei* QM6a to degrade, as indicated by stunted growth (Fig. 2) and low hydrolyzability (Fig. 8). The initial accessibility to enzymes was comparable to that of NBSK (Fig. 1). However, because the pretreatment chemically altered lignin, but did not rearrange lignin and hemicellulose in the cell wall layers, shielding of cellulose is likely maintained as hydrolysis progresses. This effectively resulted in sustained recalcitrance and, consequently, resulted in lower sugar release and lower accumulation of proteins.

Second, the chemical composition of substrates affects overexpression of enzyme activities in cultivations of *T. reesei* on lignocellulose, which has been shown previously [8, 16, 27, 77]. NBSK, which contained the largest fraction of accessible xylan, elicited the highest specific xylanase activity (1903 U mg^−1^) and normalized on protein concentration. In turn, the mannan-rich LP-ALKOX elicited the highest volumetric and specific (6.9 U mg^−1^) mannanase activity. However, LP-STEX, in which hemicellulose was almost absent (Fig. 1), still showed a comparably high specific xylanase (1346 U mg^−1^) and mannanase activity (1.2 U mg^−1^). This suggests that other factors apart from the chemical composition, e.g., coregulation of genes or other currently unknown substrate-related factors, elicit a specific gene regulatory response in *T. reesei*, resulting in overexpression of certain enzyme classes. The onset of autophagy, for instance, has been correlated with an increased secretion of the endo-mannanase man1 [43], possibly explaining the delayed increase in mannanase-activity development on NBSK. Finally, side chains and decorations on the hemicellulose can affect gene regulatory responses [30]. Acetate, uronic acids, and sugar substitutions on hemicellulose might require removal with dedicated enzymes to provide access for backbone-acting activities (e.g., endo-xylanases and -mannanases). This might also result in delayed onsets of expression of these activities, as was observed for xylanase secretion on LP-ALKOX.

#### Adsorption pattern of enzymes in T. reesei’s secretome onto insoluble substrate

**Fig. 5:**
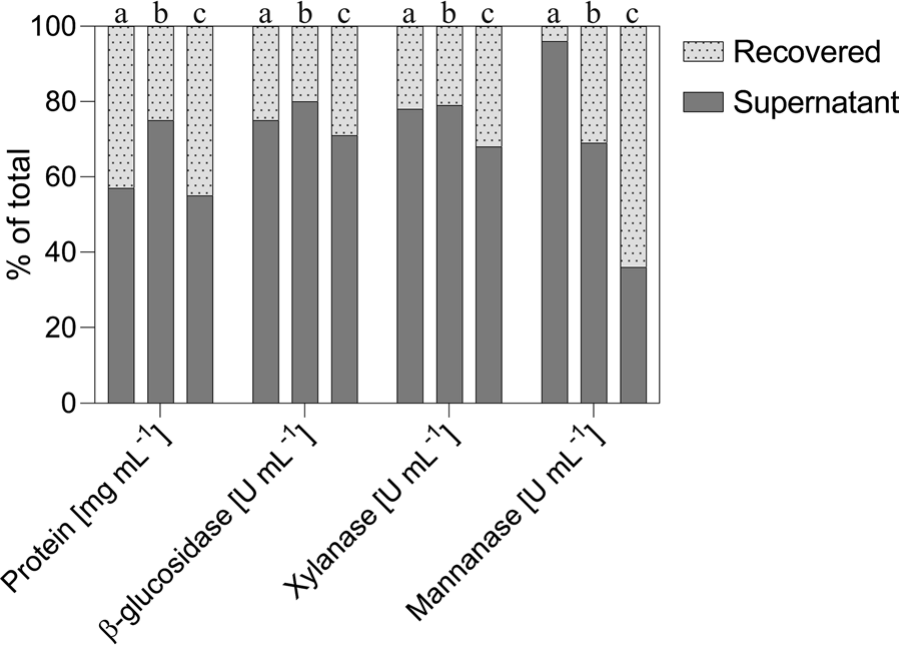
Distribution of enzyme activities between cultivation supernatant (black bars) and recovered (dotted gray bars) fraction in cultivations by *T. reesei* QM6a on NBSK (**a**), LP-STEX (**b**), and LP-ALKOX (**c**). Samples were taken after 120 h (NBSK) and 144 h (LP-STEX and LP-ALKOX) of cultivation. Desorption was performed with Tween 80, as described in Methods section. Depicted are mean values of technical triplicates

Substantial amounts of proteins (25–45%) and enzyme activities (4–64%) recovered from the cultivation broth were adsorbed onto insoluble substrates (Fig. 5) and could be recovered by desorption, thus avoiding misrepresentation of the secretome composition and hydrolytic strength of the secreted enzyme mixture. The distribution of enzyme activities between supernatant and insoluble fraction was both activity and substrate-dependent (Fig. 5). The ß-glucosidase (22–32%) and xylanase activities (20–29%) recovered from insoluble fractions were similar for all substrates (Fig. 2). In contrast, mannanase activity adsorbed on insoluble fractions drastically increased from NBSK over LP-STEX to LP-ALKOX (4, 31, and 64%, respectively). This indicates that certain classes of enzymes, such as mannanases (Fig. 5), are more prone to interact with diverse structures and composition of the lignin macromolecule. Different classes of enzymes have been shown to be particularly prone to adsorb to lignin structures [57, 78, 79]. Adding surfactants and surfactant precursors to the cultivation medium has been shown to positively affect protein production by *T. reesei* [33], prevent loss of key activities [79], and enhance enzymatic hydrolysis efficiencies by attenuating non-specific enzyme adsorption to lignin [80].

In all instances, the fraction of proteins recovered from insoluble substrates decreased over time (Additional file 1: Figure S3). The proposed underlying mechanism relates to saturation of lignocellulosic surfaces by adsorption of proteins. The continuous substrate consumption and enzyme production led to saturation of binding sites on the substrate, resulting in accumulation of free enzymes in the supernatant [81, 82]. Similarly, unspecific binding of proteins onto lignin reaches saturation. The interpretation is also affected by proteins irreversibly bound onto and deactivated by lignin [57, 83], which results in activities that were non-recoverable by the surfactant desorption method used in this study. NBSK, which has a very low lignin content, shows a relatively small change over time, indicating that equilibria were determined by sorption behavior of carbohydrate active enzymes (Additional file 1: Figure S3). The greatest change was observed on LP-STEX, followed by LP-ALKOX (Additional file 1: Figure S3), which is attributed to unspecific adsorption onto lignin in the initial stages and subsequent saturation. Cellulases and ß-glucosidase have been shown to be particularly prone to adsorb to phenolic hydroxyl groups [78] and condensed lignin structures [57, 79] that are present in LP-STEX.

#### Activity fingerprint of the concentrated supernatants P_NBSK_, P_LP-STEX_, and P_LP-ALKOX_

**Fig. 6:**
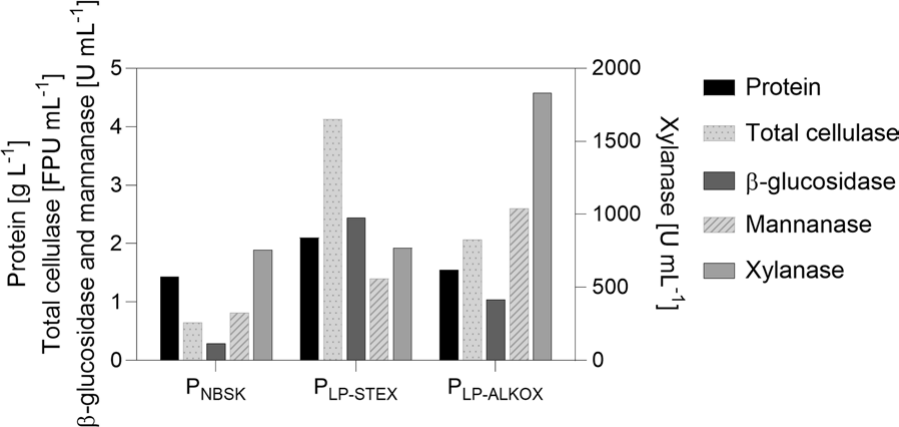
Activity and protein fingerprint of P_NBSK_, P_LP-STEX_, and P_LP-ALKOX_. Depicted are protein concentration, as well as filter paper, ß-glucosidase, mannanase, and xylanase activities in the supernatants, harvest, and concentrated as described in the methods section. Data represent mean values of technical triplicates

After 240 h of cultivation, the supernatants were harvested and concentrated and then used for secretome analysis and testing of their hydrolytic strengths, as shown hereinafter. The *T. reesei* proteins were denoted P_NBSK_, P_LP-STEX_, and P_LP-ALKOX_ according to the substrate the fungus was cultivated on. The protein concentration and enzyme activities in P_NBSK_, P_LP-STEX_, and P_LP-ALKOX_ are depicted in Fig. 6.

#### Secretome analysis of carbohydrate active enzymes

**Fig. 7:**
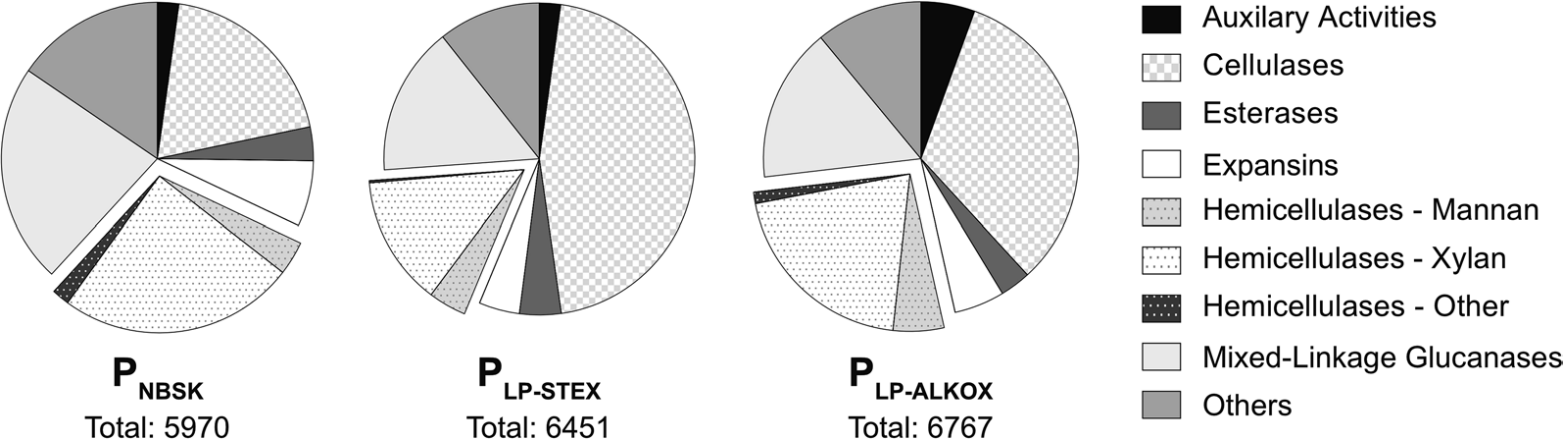
The functional distribution of CAZymes detected in the secretomes. The hemicellulases are highlighted and subcategorized according to the backbone of the hemicellulose they are degrading and contain both backbone and side-chain-cleaving enzymes. The mixed-linkage glucanases (e.g., endo-ß-1,3(/1,4)-glucanase, α-glucosidases) contain ambiguous GHs that could either not be exclusively added to one of the hemicellulases categories, or have been shown to act on hemicelluloses that are prevalent in other plants (e.g., grains [84]) and thus are not expected to be found in the softwood substrates [84]. The “others” category contained GHs active on glycoproteins, in internal carbohydrate signaling, in fungal cell wall degradation (i.e., chitinase), and enzymes with unidentified function. Detailed information is available in the Additional file 2

To further our understanding on how the softwood substrate characteristics affect fungal gene regulation, the secretome monocomponent composition was analyzed in P_NBSK_, P_LP-STEX_, and P_LP-ALKOX_. The different carbohydrate active enzymes (“CAZymes”) and families found in the respective secretomes are summarized in Table 1 and related to the total number found in *T. reesei* (TRIRE2 data base; https://mycocosm.jgi.doe.gov). Furthermore, the distributions of identified CAZymes for the different substrates, sorted according to their functionalities, are shown in Fig. 7. Detected proteins and enzymes and their abundance are detailed in Supplementary Information Secretome Data (Additional file 2).

**Table 1:** Number of CAZy enzymes and families in the secretome of *T. reesei* cultivated on NBSK, LP-STEX, and LP-ALKOX^**a**^

	CAZy enzymes		CAZy families	
	#of genes	% of TRIRE2^**b**^	#of families	% of TRIRE2^**b**^
P_NBSK_	99	39	49	69
P_LP-STEX_	86	34	45	63
P_LP-ALKOX_	81	32	42	59
On at least one substrate^**a**^	105	43	50	70

^**a**^CAA, CE, EXPN, and GH; *cf* https://mycocosm.jgi.doe.gov and SI

^**b**^Related to TRIRE2 database containing, respectively, 253 and 71 CAZyme genes and families

In total, 99, 86, and 81of 253 TRIRE2-CAZy enzymes, belonging to 49, 45, and 42 different GH, CE, AA, and EXPN families, were found in P_NBSK_, P_LP-STEX_, and P_LP-ALKOX_, respectively. Roughly, 43% of TRIRE2-CAZy genes and 70% of TRIRE2-CAZy families were expressed on at least one of the softwood substrates. This is comparable to a previous study that compared *T. reesei* secretomes from cultivations on Avicel and STEX-treated spruce, where the cellulosic model substrate Avicel (58 genes, 31 families) also elicited a higher amount of CAZymes than STEX-treated spruce (51 genes, 31 families) [30].

Inspection of Fig. 7 shows that the relative abundance of cellulolytic enzymes was highest in P_LP-STEX_ followed by P_LP-ALKOX_, and P_NBSK_, with the abundance of hemicellulolytic enzymes following the opposite trend. Relative to their contribution, enzymes belonging to the mannan- and xylan-degrading machinery were highest in P_LP-ALKOX_ and NBSK-derived secretomes, respectively. Thus, secretome results follow and support trends observed in the cultivation time courses, where NBSK and LP-ALKOX elicited the highest specific xylanase and mannanase activities, respectively. A more detailed secretome analysis (Additional file 2) showed that the cellulolytic “work horse” cellobiohydrolase Cel7a was most abundant in all three cases. The ten most abundant entries contained additional enzymes of the cellulose-degradation machinery, including Cel6a, endoglucanases, and ß-glucosidases. Interestingly, all secretomes contained a large amount of Swo1. Although this expansin-like protein has previously been described to be overexpressed [8, 30, 44], its function in cellulose degradation is still debated.

To derive insights into differences among the three secretomes, differential expression of genes encoding for CAZymes was quantified. CAZymes showing the largest variation in abundance (log2-fold change > 2) are summarized in the Additional file 2. In accordance with time courses and Fig. 7, NBSK triggered a significant upregulation of xylan-degrading enzymes (GH3, GH16, and GH30) and LP-ALKOX elicited a higher abundance of mannan-degrading enzymes (GH2, GH92).

Interestingly, P_LP-STEX_ and P_LP-ALKOX_ compared similarly to P_NBSK_, with 8 out of 14 significantly more abundant proteins being the same (Additional file 2), despite differences in substrates’ characteristics (Fig. 1). As mentioned before, this suggests that other factors are at play to affect the gene regulatory machinery. Some of the proteins, e.g., lysozyme (GH25), chitinase (GH18), and α-1,4-mannosidase (GH92), have been connected to autophagy in the previous studies [43]. It is worth mentioning that secretome data represent the end point of the cultivations, thus, genes typically found in conjunction with starvation are expected to start accumulating on all substrates. Because the two high-lignin substrates, LP-STEX and LP-ALKOX, create the additional hurdle of increasing lignin to carbohydrate ratios, these enzyme groups could have started to accumulate earlier or faster. However, as summarized in Fig. 7, the mixed-linkage glucanases category is actually higher in P_NBSK_, and no variation can be observed in the “other” CAZymes (containing many chitinases and glyco-protein specific enzymes, Additional file 2). This implies that upregulation is on specific enzymes rather than their overall function. Alternatively, coregulation of genes on complex substrates has been suggested [44], which might be triggered by certain aspects (e.g., lignin or lignin–hemicellulose complexes) of complex substrates. For an instance, additional glucanases (e.g., GH12, GH64) might be required to overcome decreased accessibility. A slightly larger fraction of esterases produced on complex substrates (Additional file 2), in particular on LP-ALKOX (Fig. 7), might imply that the fungus tries to overcome recalcitrance caused by a heavy decoration of hemicellulosic side chains and by linkages between hemicellulose and lignin (lignin–carbohydrate complex or LCC bonds) [85].

Two additional enzymes that are more abundantly expressed on LP-ALKOX than on the two other substrates belong to the AA9 family—the lytic polysaccharides monooxygenases (LPMO, Additional file 2). These oxidative enzymes have been shown to act on crystalline cellulose as well as various hemicelluloses [86], boosting enzymatic hydrolysis efficiencies drastically [87]. This effect has been shown on several industrially relevant substrates [88–90]. However, *T. reesei* only harbors 3 AA9 genes (cf, Additional file 2), much fewer than other filamentous fungi that have 10–20 different LPMOs [91]. Indeed, the relative abundance of the two LPMOs identified in this study was very low (cf Additional file 2), which is in agreement with a previous study [91]. Hence, further analysis of the activity of LPMOs and its effect on the enzymatic hydrolysis efficiency was not included in this study and enzymatic hydrolyzes were not run under conditions promoting LPMO activity [86].

### Supplementation of a cellulolytic enzyme mixture with “tailored” *T. reesei* supernatant increases hydrolytic efficiency

**Fig. 8:**
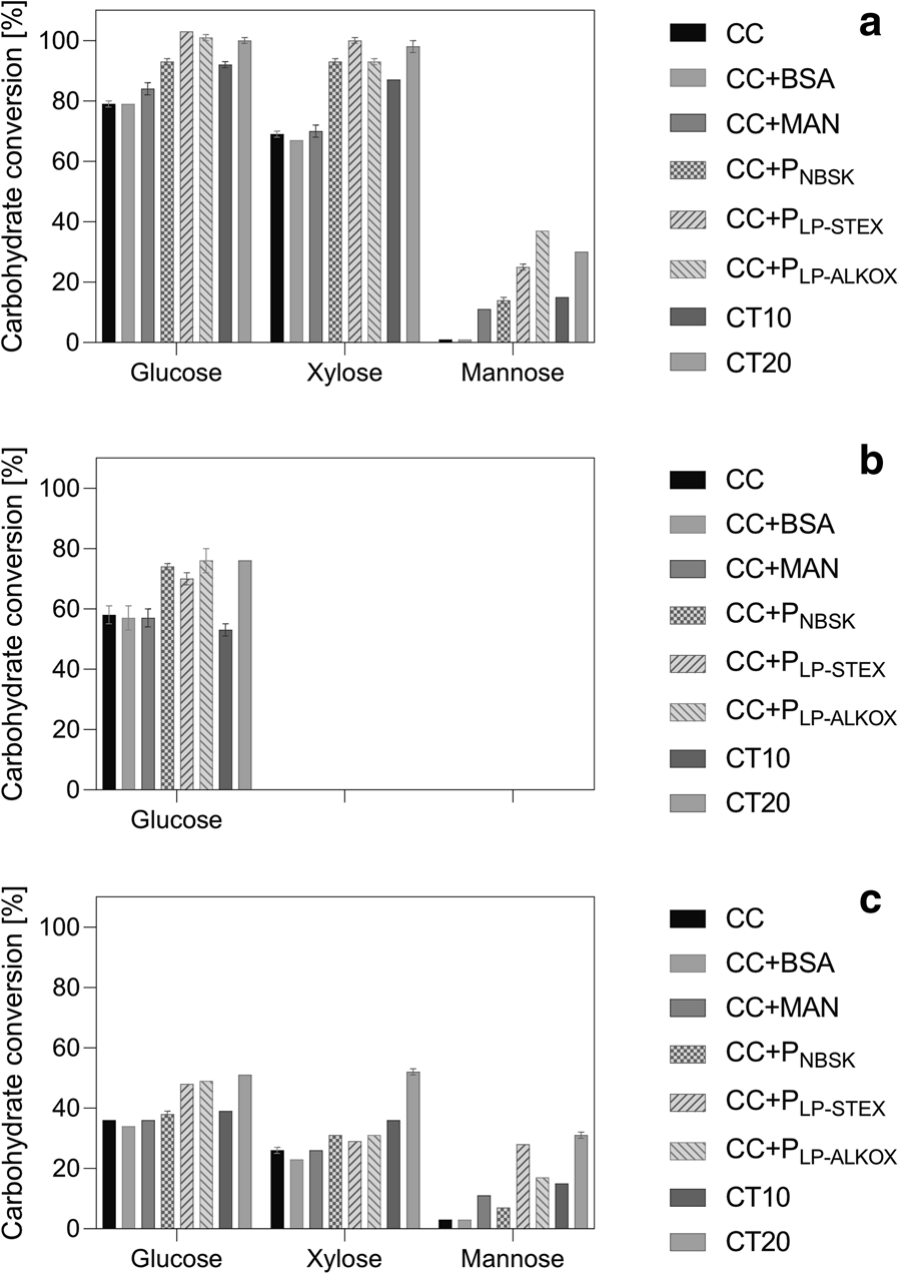
The effect of *T. reesei* enzyme mixtures on enzymatic hydrolysis performance. Depicted are conversion yields for enzymatic hydrolysis of NBSK (a), LP-STEX (b), and LP-ALKOX (c) with Celluclast (CC) and Celluclast supplemented with BSA (CC + BSA), endo-mannanase (CC + MAN), and concentrated *T. reesei* QM6a cultivation supernatants (CC + P_NBSK_, CC + P_LP-STEX_, and CC + P_LP-ALKOX_). All reactions were run at a CC protein loading of 20 mg g^−1^ substrate dry mass. Supplementation of fungal protein, BSA, and mannanase was at a protein loading of 5 mg g^−1^ substrate dry mass, as detailed in Methods section. As benchmark, hydrolysis reactions with Cellic Cetc3 at 10 (CT10) and 20 (CT20) FPU g^−1^ dry mass are also depicted. All data represent mean values from duplicate experiments and error bars represent the spread

The hydrolytic strength of P_NBSK_, P_LP-STEX_, and P_LP-ALKOX_ to hydrolyze the three softwood substrates used in this study was tested and compared by augmenting it with the basic cellulosic enzyme cocktail Celluclast (CC). The underlying aim was to mimic a minimal enzyme cocktail where the core cellulolytic enzymes (endo- and exo-glucanases, ß-glucosidases) are supplemented with enzyme mixtures naturally adapted by the fungus to overcome specific substrates’ recalcitrance. Apart from CC supplemented with P_NBSK_, P_LP-STEX_, and P_LP-ALKOX_ hydrolyzes were performed with CC alone (base case), and with CC supplemented with BSA and commercial mannanase. BSA addition is a control to exclude the effect of additional protein on hydrolysis yields, whereas commercial mannanase supplementation was used to evaluate the drastic improvements of mannan conversion observed by *T. reesei* protein addition, as discussed hereinafter. Finally, we benchmarked our enzyme system with the state-of-the art preparation Cellic Ctec3. The results are depicted in Fig. 8.

The hydrolyzability of substrates in the base case decreased (CC; Fig. 8) with increasing structural and chemical complexity (NBSK > LP-STEX > LP-ALKOX). Blocking of unspecific binding sites with BSA (CC + BSA) had a marginal effect on conversion efficiencies. This shows that improved hydrolysis yields were not caused by additional protein preventing unspecific binding, indicating that losses of key activities were insignificant.

Supplementation with fungal enzyme mixtures (CC + P_NBSK_, CC + P_LP-STEX_, and CC + P_LP-ALKOX_) resulted in 6–27% increase in cellulose conversion, reaching full conversion of the cellulose component in P_LP-STEX_ and P_LP-ALKOX_ supplemented hydrolysis of NBSK. Significant improvement was also achieved for xylan and mannan conversion in NBSK (34–45% and 955–2737%, respectively) and LP-ALKOX (12–18% and 121–737%, respectively). Hemicellulose conversion for LP-STEX is not shown because of the marginal hemicellulose content in the substrate (Fig. 1), which makes accurate yield calculations difficult.

Interestingly, mannan conversion in reactions supplemented with P_LP-STEX_ and P_LP-ALKOX_ drastically exceeded those supplemented with commercial mannanase activity (CC + MAN), with improvements of 131–251% and 52–146% on NBSK and LP-ALKOX, respectively. This is striking, considering that mannanase loading in the reactions (3.3 and 8.2 U g^−1^ for P_LP-STEX_ and P_LP-ALKOX_, respectively) was much lower than in the commercial preparation (1722 U g^−1^). This clearly highlights that specific enzymes in addition to backbone-cleaving hemicellulases, e.g., mannanases as shown here, are required to overcome the recalcitrance posed by the softwood hemicellulose. Connecting back to the secretome data, these specific enzymes likely comprised of side-chain-cleaving activities, such as α-galactosidases, α-glucoronidases, α-L-arabinofuranosidases, or acetylxylanesterases (Fig. 7, Additional file 2).

The enhancement for xylan and mannan degradation was accompanied by improved efficiency of cellulose hydrolysis. This was likely a result of more efficient removal of the hemicellulose shield [56, 92], as well as an inherent difference in cellulolytic enzyme loadings (12.2 FPU g^−1^ (CC) + 2.2, 9.6, and 6.6 FPU g^−1^ in P_NBSK_, P_LP-STEX_, and P_LP-ALKOX_, respectively). The effect of enhanced hemicellulose degradation on cellulose hydrolysis was most pronounced with concentrated supernatants derived from cultivation on complex substrates (P_LP-STEX_ < P_LP-ALKOX_).

Finally, we benchmarked results against the state-of-the art commercial enzyme cocktail Cellic Ctec3 (denoted “CT”, Fig. 7). The experiment was designed to bracket enzyme loadings achieved by CC plus *T. reesei* protein. Due to the drastically higher activity-to-protein ratio in CT as compared to our enzyme mixture, this was done based on filter paper activity (10 and 20 FPU g^−1^ dry mass substrate; denoted CT10 and CT20, respectively). Surprisingly, on NBSK, CC supplemented with either P_LP-STEX_ or P_LP-ALKOX_ exceeded cellulose, xylan, and mannan conversion of that obtained with CT20. On LP-STEX and LP-ALKOX, cellulose conversion yields with CT20 were at par, as was xylan conversion on LP-ALKOX. Only mannan conversion on LP-ALKOX was superior using CT20.

These results clearly show that supplementation of a basic cellulolytic enzyme cocktail with P_LP-STEX_ and P_LP-ALKOX_ resulted in excellent hydrolysis yields, exceeding the base-case scenario and controls (CC, CC + BSA, CC + MAN) over the full range of substrates. Furthermore, when benchmarking against state-of-the-art enzyme cocktails, the supplemented basic cellulolytic enzyme cocktails were more or similarly efficient. This strongly supports the notion that addition of tailored and specific enzymes is essential for degradation of complex lignocellulosic substrates [15, 16, 25, 35]. These enzymes and proteins can be produced by *T. reesei* without a priori knowledge of required activities by cultivation on feedstock used in the biorefinery, as suggested before and supported by techno-economic considerations [8].

## Conclusion

This study shows that substrate characteristics can be directly linked to fungal growth, gene regulation, and protein expression in *T. reesei* cultivations. Specifically, the hemicellulose content and composition, accessibility, and substrate complexity impacted the temporal development of enzyme activities and the secretomes’ monocomponent composition produced by *T. reesei* QM6a. CLSM imaging further showed substrate-dependent changes in fungal micromorphology, which were likely caused by differences in sugar release rates (accessibility and complexity) and mass transfer limitations by cultivation broth rheology (surface charge and fiber dimensions). By facilitating differential staining of hyphae and substrate solids, CLSM images further suggested that the fungus is growing closely associated with the softwood substrates. Although these results clearly support the proposed interrelation of feedstock characteristics and the gene regulatory response in *T. reesei*, this study also revealed that some other, currently unknown, substrate factors play a role. This was mainly observed for the two complex substrates (LP-STEX and LP-ALKOX), which triggered similar upregulation of genes, despite their differences in substrate characteristics, clearly guiding future research.

Finally, we show that supplementation of a basic cellulase mixture with *T. reesei* protein harvested after cultivation improved cellulose, xylan, and mannan conversion drastically, even exceeding benchmark yields obtained with the state-of-the-art enzyme preparation, Cellic Ctec3. The most complex substrates (LP-STEX and LP-ALKOX) elicited the most dramatic improvements. Thus, we show that customized enzyme mixtures can be effectively produced by exploiting the natural adaptation potential of *T. reesei*, opening the door for future biorefinery process improvements.

## Materials and methods

### Raw material and pretreatment

Commercial northern bleached softwood Kraft pulp (NBSK), dilute acid-catalyzed steam-pretreated Lodgepole pine (LP-STEX), and mild alkali-oxygen pretreated Lodgepole pine (LP-ALKOX) were used as substrates for fungal cultivation. LP-STEX and LP-ALKOX were produced from commercial Lodgepole pine (*Pinus contorta*) wood chips. The pretreatments and properties of the materials are summarized below and are shown in Fig. 1 and are detailed in the Additional file 1 (Method S1, Table S1, and Figure S1).

In brief, sheet dried NBSK from the British Columbia interior (Canada), typically consisting of 50–60% Lodgepole pine, 30–40% White spruce, and 5–10% sub-alpine fir, was obtained from industrial sources. The sheet dried NBSK was rehydrated and disintegrated non-destructively per ISO 5263 procedures and dewatered by vacuum filtration.

The LP-STEX was prepared by dilute-acid-catalyzed steam pretreatment. Lodgepole pine wood chips were wetted to 50 wt.% dry matter, impregnated with 2 wt.% SO_2_ based on dry matter, and steam-pretreated at 210 °C for 5 min in a batch pretreatment reactor. The pretreated material was dewatered by vacuum filtration.

The LP-ALKOX was prepared by impregnation with sodium carbonate, thermochemical treatment, and mechanical refining. Wood chips were impregnated with 20 wt.% sodium carbonate solution for 12 h at 70 °C. Impregnated wood chips were suspended in a mesh basket in a 1-L Parr reactor, injected with molecular oxygen at 6.9 bar, and treated at 130 °C for 6 h. The treated wood chips were mechanically refined by twin-screw extrusion to defibrate the wood chips and develop fibers. Subsequently, shives were removed with a plate-type vibrating screen, fibers PFI-milled for 5000 revolutions at standard conditions to homogenize the fiber morphology and increase fibrillation, and the fibers dewatered by vacuum filtration.

### Substrate characterization

#### Compositional analysis

The water-insoluble solid content, dry matter content, and composition of structural carbohydrates, lignin, and inorganic ash in materials were measured in triplicates with National Renewable Energy Laboratory standard methods [93–95].

#### Fiber morphology

The distributions of fiber length and width were measured by a HiRes Fiber Quality Analyzer LDA02 (OpTest Equipment, Canada), conforming with ISO 16,065. Fibers were suspended in distilled water and diluted to yield fiber counts between 30 and 50 s^−1^ during analysis. Sample size was 10,000 fibers and fiber length measurement limits were set to 0.05 and 10 mm. Fibrous particles < 200 µm in length were defined as fines.

#### Total acidic group content

The total acidic group contents of the pretreated materials were measured in triplicates by conductometric titration using the Scandinavian Pulp, Paper and Board Testing Committee test method SCAN-CM 65:02.

#### Cellulose accessibility to enzymes

The accessibility of cellulose to enzymes was assessed per modified Simons’ staining method [96], using the high-molecular-weight fraction of Direct Orange 15 (Pylam Products, USA) that has high affinity for cellulose. The preferential adsorption of dye onto cellulose in pretreated materials provide the basis for comparing the relative accessibility of cellulose to enzymes, using maximum monolayer capacity, derived from the linearized Langmuir adsorption isotherm, as a proxy for accessibility.

#### Water retention value

The water retention values (WRV) of substrates were measured in triplicates using a modified version of the Scandinavian Pulp, Paper and Board Testing Committee test method SCAN-C 62:00 [97].

### Enzyme production by *T. reesei*

#### Fungal cultivation

Cultivations were performed with the fully sequenced wild-type-like *T. reesei* QM6a strain. A detailed description of the cultivation strategy is given in the Additional file 2 (Method S2). In brief, fungal cultivations were performed in bioreactors with 2 L working volume, using mineral medium with 15 g L^−1^ of the respective softwood substrates, based on dry matter. Each cultivation was inoculated with two pooled 50-mL precultures, consisting of mineral medium supplemented with 10 g L^−1^ lactose. The cultivations were performed and controlled at 28 °C, pH 5, and 20% dissolved oxygen, for 200-h total cultivation time.

#### Sample preparations

Samples (≈5 mL) were taken regularly, from which 1.8 mL was immediately fixated with 200-µL 10 vol.% formaldehyde solution and stored at 4 °C awaiting imaging. Furthermore, 2 mL of sample was centrifuged (13,000 × *g*, 2 min) and the supernatant recovered and stored at 4 °C awaiting protein concentration and enzymatic activity measurements. Additionally, enzymes and proteins bound to insoluble substrates were recovered by mixing the remaining solids with 1 mL of 1 wt.% Tween 80 in 20 mM sodium acetate buffer, pH 4.8, and incubating the mixture at 45 °C under agitation for 2 h. The supernatant was recovered by centrifugation (13,000 × *g*, 2 min) and stored at 4ºC awaiting analysis.

#### Preparation of cultivation supernatant for enzymatic hydrolysis

After 240 h of cultivation, the supernatant was harvested by vacuum filtration and enzymes and proteins bound to insoluble substrate recovered by washing with Tween 80 solution, as described above. The two fractions were pooled, washed with 20 mM sodium acetate buffer (pH 4.8), and the original volume reduced tenfold using an Amicon 8400 protein concentrator (Merck-Millipore, USA) with a 5 kDa cut-off PES membrane. The washed and concentrated supernatants, denoted P_NBSK_, P_LP-STEX_, and P_LP-ALKOX_, respectively, were stored at 4 °C.

#### Enzymatic activity and protein measurements

Protein concentration was measured with the Bradford method [98], with prior protein precipitation. Filter paper activity (FPU) was measured following IUPAC recommendations [99]. β-Glucosidase, xylanase, and mannanase-activity measurements were performed as described elsewhere [100], with the release of reducing sugars from xylan and glucomannan quantified with the DNS (3,5-dinitrosalicylic acid) method [99]. All measurements were performed in duplicates. A detailed description of the assays is given in the Additional file 2: Method S3.

#### Secretome analysis by nanoLC–MS/MS

Proteins in P_NBSK_, P_LP-STEX_, and P_LP-ALKOX_ were precipitated with trichloroacetic acid, washed extensively, and then denaturated and tryptically digested. After sample clean-up (OMIX C18 SPE cartridges, Agilent, USA), peptides were analyzed by nanoLC–MS/MS using an Agilent 1100 nanoflow system (Agilent) connected to hybrid linear ion trap–orbitrap mass spectrometer (LTQ-Orbitrap Elite™, Thermo Fisher Scientific, USA) equipped with an EASY-Spray™ electrospray source (held at constant 35 °C). Chromatography of peptides prior to mass spectral analysis was accomplished using a capillary emitter column (PepMap® C18, 3 µM, 100 Å, 150 × 0.075 mm, Thermo Fisher Scientific, USA).

Raw MS/MS data files were searched using the Mascot search engine (ver. 2.2.07) against Treesei_JGI_proteins.FilteredModelsV2.0_REV_CP_2019 database (https://mycocosm.jgi.doe.gov/Trire2/Trire2.home.html). A detailed description of sample preparation, nanoLC-MS/MS analysis, and data analysis is provided in the Additional file 2: Method S4.

#### CLSM imaging

Calcofluor white stain (denoted “CF”; #18,909, Sigma-Aldrich, USA), wheat germ agglutinin-AlexaFluor488 dye conjugate (“AF”; #W11261, ThermoFisher Scientific, USA), and FM 5–95 (“FM”; #T23360, ThermoFisher Scientific, USA) were used as dyes. The fixed samples were dyed as recommended by the manufacturer, mounted, and sealed with nail polish. Confocal laser scanning microscopy (CLSM) imaging was performed with a Zeiss 710 LSM (Carl Zeiss AG, Germany) with a 40x, 1.2NA objective. Dyes, excitation wavelengths, and emission collection ranges were chosen to maximize the contrast between the hypha and fiber. The excitation/emission wavelengths used were: CF: 405/424–502 nm, AF: 488/499–608 nm, and FM: 488/499–591 nm. For morphology investigation, CF only was used. To analyze interaction between hypha and fiber, the maximal contrast was achieved by the following dye combinations for staining hyphae/solids: NBSK: AF/CF, LP-STEX:CF/AF, and LP-ALCOX:CF/FM. Z-stacks of 14–39 optical slices at 2-µm intervals were obtained and 2D renditions were created by maximum intensity projection in Fiji [101]. Typically, 2–6 images on biological replicates were taken, and representative samples shown herein.

### Enzymatic hydrolysis of softwood substrates with supplemented enzyme mixtures

Enzymatic hydrolysis reactions were performed in 2-mL screw cap tubes with 1.5-g total reaction weight. Substrate loadings of 1.5 wt.% dry matter in 20 mM sodium acetate buffer, pH 4.8, were used. In the base case, reactions were performed with Celluclast (Novozymes A/S, Denmark; total cellulase: 44 FPU mL^−1^; protein: 72 mg mL^−1^; ß-glucosidase: 52 U mL^−1^; xylanase: 3396 U mL^−1^; mannanase: 4 U mL^−1^) at a protein loading of 20 mg g^−1^ of substrate dry matter. Furthermore, the base case was supplemented with P_NBSK_, P_LP-STEX_, and P_LP-ALKOX_ at protein loadings of 5 mg g^−1^ of substrate dry matter. As controls, the base case was supplemented with BSA or endo-mannanase activity from *Aspergillus niger* (mannanase activity: 47,330 U mL^−1^, protein: 74 mg mL^−1^) at a protein loading of 5 mg g^−1^ of substrate dry matter. As benchmarks, hydrolysis reactions were further run with Cellic Ctec3 (Novozymes A/S, Denmark; total cellulase: 170 FPU mL^−1^; protein: 110 mg mL^−1^). Because of the much higher FPU-to-protein ratio of Ctec3 as compared to Celluclast, the enzyme loading was based on activity at 10 and 20 FPU g^−1^ dry matter, representing a protein loading of ~ 7 and ~ 14 mg g^−1^ dry matter, respectively. The reactions ran for 48 h at 50 °C in a thermoblock and mixed by manually inverting the tubes regularly. To stop the reaction, mixtures were brought to 100 °C for 10 min and then stored at -20 °C awaiting analysis.

### Analytical methods

Monomeric sugars were measured by isocratic high-performance anion-exchange chromatography with pulsed amperometric detection (ICS-3000, ThermoFisher Scientific, USA), using a CarboPac PA1 guard and analytical column (ThermoFisher Scientific). Measurements were performed isocratically at 30 °C with deionized water as mobile phase at a flow rate of 1 mL min^−1^.

## Supplementary Information

**Additional file 1.** Supplementary information containing Supplementary Methods S1 to S5, Supplementary Tables S1 and S2, and Supplementary Figures S1 to S3.

**Additional file 2.** Secretome raw data and substrate-dependent differences in protein abundancy. Data processing include the 10 most abundant proteins in each secretome, significant (log2>2) substrate-dependent changes in protein abundancy, and assignment of proteins to their CAZy families.

## Data Availability

All data generated or analyzed during this study are included in this published article and its supplementary information files.
