# Supplementary material for: The characteristics of insoluble softwood substrates affect fungal morphology, secretome composition, and hydrolytic efficiency of enzymes produced by Trichoderma reesei
Source: Biotechnol Biofuels. 2021 Apr 26;14:105. doi: 10.1186/s13068-021-01955-5 (PMC8074412; doi:10.1186/s13068-021-01955-5)
Supplement: Supplementary file 1 — Additional file 1. Supplementary information containing Supplementary Methods S1 to S5, Supplementary Tables S1 and S2, and Supplementary Figures S1 to S3. [file 13068_2021_1955_MOESM1_ESM.pdf]

Supplementary Information

The characteristics of insoluble softwood substrates affect fungal morphology, secretome composition, and hydrolytic efficiency of enzymes produced by *Trichoderma reesei*

Vera Novy[1,†,‡,§], Fredrik Nielsen[1,†,‡], Daniel Cullen[1], Grzegorz Sabat[2], Carl J. Houtman[1], and Christopher G. Hunt[1]

[1] US Department of Agriculture, Forest Products Laboratory, One Gifford Pinchot Drive, Madison, Wisconsin 53726, USA.

[2] University of Wisconsin Biotechnology Center, Madison, Wisconsin 53706, USA.

[†] Present address: Chalmers University of Technology, Department of Biology and Bioengineering, Division of Industrial Biotechnology, Kemivägen 10, SE-412 96 Göteborg, Sweden.

[‡] Equally contributing first authors

[§] Corresponding author: E-mail: vera.novy@t-online.de

Supplementary material contains:

Supplementary Method(s): Methods S1 to S5

Supplementary Table(s): Tables S1 to S2

Supplementary Figure(s): Figures S1 to S3

Supplementary Methodology – S1

Detailed description of pretreatment methods

Three substrates were prepared: northern bleached softwood Kraft pulp (NBSK), dilute acid-catalyzed steam pretreated Lodgepole pine (LP-STEX), and mild alkali-oxygen pretreated Lodgepole pine (LP-ALKOX). Substrates from Lodgepole pine (*Pinus contorta*) were produced from commercial Lodgepole pine (LP) wood chips with a humidity content of 8.0±0.6 wt.%. The wood chips were stack-sieved and the 16×16 to 31.5×31.5 mm size fraction with an average thickness of 4.1 mm was used.

Sheet dried NBSK from the British Columbia interior (Canada), typically consisting of 50-60% Lodgepole pine, 30-40% White spruce, and 5-10% sub-alpine fir, was obtained from industrial sources. The sheets were soaked in deionized water for 60 min and disintegrated with 30 000 revolutions in a standard disintegrator at 1 wt.% consistency, conforming with ISO 5263. The dispersed fibers were dewatered by vacuum filtration and stored at 4°C.

For LP-STEX, LP wood chips were impregnated with water at 30°C overnight at a wood to liquid mass ratio of 1, yielding a dry matter (DM) content in the wood chips of ≈50 wt.%. The wood chips were subsequently impregnated with gaseous SO_2 in sealed plastic bags for 20 minutes at room temperature and vented for 30 min, absorbing 2 wt.% of SO_2, based on DM. The impregnated wood chips were steam pretreated in batches of 500 g DM at 210°C for 5 min in a preheated 10-L batch pretreatment reactor. The pretreated material was dewatered by vacuum filtration and stored at 4°C.

For LP-ALKOX, LP wood chips were autoclaved (121°C, 20 min) to displace entrained air and impregnated with 20 wt.% sodium carbonate solution, wood to liquid mass ratio 1:4, for 12 h at 70°C in a water bath. Impregnated wood chips were placed in a mesh basket, suspended above the liquid level, in a 1-L stainless steel-lined Parr reactor (Parr Instrument Company, Moline, IL, USA) and injected with molecular oxygen (grade 5.0) at 6.9 bar. The impregnated wood chips were treated at 130°C for 6 h with a rise time of 45 min from 70°C to treatment temperature. Mechanical refining was performed in a 3-step process: defibration, screening, and PFI-milling. The treated wood chips were suspended in ≈5 L of water and mechanically refined by twin-screw extrusion, using a commercial juicer (Angel model 8500), to defibrate the wood chips and develop fibers, and subsequently dewatered by filtration and washed with ≈15 L of deionized water. Shives were removed with a plate-type vibrating screen with

0.3048×45 mm slits and the fibers collected on a 100-mesh screen (0.149×0.149 mm). Collected fibers were PFI-milled for 5000 revolutions with a beating gap of 0.2 mm at 10 wt.% consistency to homogenize the fiber morphology and increase the fibrillation. The pretreated material was dewatered by vacuum filtration and stored at 4°C.

Supplementary Methodology – S2

Fungal bioreactor cultivations

T. reesei QM6a was kept on potato dextrose agar and revitalized every 2 weeks by transferring a piece of overgrown agar to a fresh agar plate. Incubation was at 30ºC for 1 week. Pieces of overgrown agar was then used to inoculate two pre-cultures consisting of 50 mL of mineral medium (5 g L^{-1} KH_2PO_4, 5 g L^{-1} yeast extract, 3.75 g L^{-1} $(NH_4)_2SO_4$, 0.3 g L^{-1} $MgSO_4 \times 7\ H_2O$, 0.3 g L^{-1} $CaCl_2 \times 2\ H_2O$, and 1 mL L^{-1} of trace element solution, containing 5 g L^{-1} $FeSO_4 \times 7\ H_2O$, 1.6 g L^{-1} $MnSO_4 \times 1\ H_2O$, 0.67 g L^{-1} $ZnCl_2$, 2 g L^{-1} $CoCl_2 \times 6\ H_2O$, and 15 g L^{-1} EDTA-Na_2) supplemented with 10 g L^{-1} lactose in 125-mL Erlenmeyer flasks closed with a foam stoppers. Incubation was at 28ºC and 190 rpm on an orbital shaker for 3 days.

Bioreactor cultivations were performed in an Applikon bioreactor system comprising an ADI-1025 BioConsole, an ADI-1010 BioController, and a 3.2-L glass fermentation vessel (Applikon Biotechnology, Delft, the Netherlands), using a 2 L working volume. The two precultures were pooled and added to the bioreactor, which contained mineral medium supplemented with 15 g L^{-1} of the respective softwood substrate, based on dry matter. The cultivations were run at 28°C, pH 5, and 20% dissolved oxygen, based on air saturation. Temperature was controlled by a heating blanket and an internal cooling finger. pH was controlled with automatic addition of 1 M KOH. Agitation was provided by two 6-blade turbine impellers and the reactor was aerated at a constant rate of 1 L min^{-1} by a pipe sparger with a porous nozzle. DO was controlled by closed-loop control of the agitation rate (range: 200-500 rpm).

Supplementary Methodology – S3

Measurement of protein concentration and enzymatic activities

To avoid interference with other compounds secreted by the fungi, the proteins in 200 µL appropriately diluted sample were precipitated prior to protein quantification with 20 µL of 500 mM KH_2PO_4 (pH 7.4) and 20 µL 250 mM $CaCl_2$. After washing the pellet with 500 µL absolute ethanol, the pellet was resuspended in 200 µL concentrated Bradford reagent (#5000006, Bio-Rad, Hercules, CA, USA). After 10 min incubation, the solution was diluted with 800 µL of filtered deionized water and the protein concentration measured at 595 nm against BSA standards.

β-Glucosidase and xylanase activity measurements were conducted as end-point microtiter assays with 200 µL reaction volume at 50°C and pH 5 (50 mM sodium citrate buffer; Na-Ci-50) for 5-10 min. The substrate for the β-glucosidase activity measurement was 0.5 mM para-nitrophenyl-*β*-d-glucopyranoside, the reaction was stopped with 1 M of Na_2CO_3, and the microtiter plate read at 405 nm. The substrate solution for the xylanase assay was prepared by dissolving 12 g L^{-1} birchwood xylan in Na-Ci-50, bringing it to a boil under agitation. Then, 150 µL of the substrate solution was mixed with 50 µL appropriately diluted enzymes, incubated for 5 min, the released sugar analyzed with the DNS method, and the microtiter plates read at 540 nm.

Endo-1,4-β-mannanase activity was measured in 400 µL reaction volume as end-point assay at 50°C and pH 5.5 (100 mM sodium citrate buffer, Na-Ci-100). The substrate solution was prepared with 2.5 g L^{-1} locust bean gum in Na-Ci-100, prepared as described for birchwood xylan. Then, 380 µL was mixed with 20 µL appropriately diluted enzymes, incubated for 5 min, the released sugar analyzed with the DNS method, and the microtiter plates read at 540 nm.

Supplementary Methodology – S4

Nano-LC-MS/MS analysis

Sample preparation

Samples for LC-MS/MS analysis were prepared by protein precipitation with trichloroacetic acid (TCA). In brief, 100 mg mL^{-1} of solid TCA was added to filtered cultivation supernatants, mixed, and frozen at -20°C over-night. After centrifugation, the precipitated proteins were washed 3 times with ice cold acetone and dried.

Milligram quantities of the TCA/acetone precipitated cultivation supernatants were solubilized in SDS-buffer (1% SDS and 2 mM EDTA in 20 mM Tris-HCl, pH 7.5) to 3 mg mL^{-1} final protein concentration from which 35 µL (equaling 105 µg of protein) were taken for downstream digestion. Proteins were again precipitated with four volumes excess of acetone. For complete protein denaturation prior to the digestion step, 30 µL of precipitation buffer (8M urea and 1 mM Tris-HCl in 50 mM NH_4HCO_3, pH 8.5) were added to the pellets and samples were incubated overnight at ~6°C. Subsequently, the samples were diluted with 5 µL of 25 mM DTT, 10 µL methanol and 75 µL of 25 mM NH_4HCO_3 (pH 8.5) and incubated at 52°C for 15 minutes. After cooling the mixture on ice to room temperature, 6 µL of 55 mM chloroacetamide was added for alkylation and incubated in darkness at room temperature for 15 minutes. Reaction was quenched by adding 16 µL of 25 mM DTT. Finally, 16 µL of a Trypsin/LysC solution (100 ng μL^{-1} Trypsin from Promega; LysC from FujiFilm-mix in 25 mM NH_4HCO_3) and 42 µL of 25 mM NH_4HCO_3 (pH 8.5) were added to 200 µL final volume. Digestion was conducted for 2 h at 42 °C, when an additional 8 µL of Trypsin/LysC solution was added and digestion proceeded overnight at 37°C. Reaction was terminated by acidification with 2.5 % TFA (Trifluoroacetic Acid) to a 0.3 % final concentration.

NanoLC-MS/MS

Digests were cleaned up using OMIX C18 SPE cartridges (Agilent) per manufacturer protocol, eluted in 20 µL of TFA (0.1 % TFA in 50 % acetonitrile and 50 % H_2O), and dried to completion in the speed-vac and finally reconstituted in 100 µL of 0.1 % formic acid. Peptides were analyzed by a nanoLC-MS/MS using the Agilent 1100 nanoflow system (Agilent) connected to hybrid linear ion trap-orbitrap mass spectrometer (LTQ-Orbitrap Elite™, Thermo Fisher Scientific) equipped with an EASY-Spray™ electrospray source (held at constant 35 °C). Chromatography of peptides prior to mass spectral analysis was accomplished using a

capillary emitter column (PepMap® C18, 3 μM, 100 Å, 150 x 0.075 mm, Thermo Fisher Scientific) onto which 0.5 and 3 μL of extracted peptides was automatically loaded. NanoHPLC system delivered solvents A (0.1 % (v/v) formic acid) and B (99.9 % (v/v) acetonitrile, 0.1 % (v/v) formic acid) at 0.50 μL min^{-1} to load the peptides (over a 30 min period) and 0.3 μL min^{-1} to elute peptides directly into the nano-electrospray with a the following gradients: 0 % (v/v) B to 30 % (v/v) B gradual over 155 min and 30-50 % B fast gradient for 10 min, and 50-95 % (v/v) for a 7 min flash-out. As peptides eluted from the HPLC-column and the electrospray source, survey MS scans were acquired in the Orbitrap with a resolution of 120,000 followed by MS2 fragmentation of the 20 most intense peptides detected in the MS1 scan from 300 to 2000 m/z; redundancy was limited by dynamic exclusion.

Data analysis

Raw MS/MS data files were searched using the Mascot search engine (ver. 2.2.07) against Treesei_JGI_proteins.FilteredModelsV2.0_REV_CP_2019 database (18,334 forward and decoy reverse entries) appended with sequences of the common lab contaminants. Variable carbamidomethylation, methionine oxidation with asparagine and glutamine deamidation, 2 tryptic miss-cleavages and peptide mass tolerances set at 15 ppm with fragment mass at 0.6 Da were selected. Peptide and protein identifications were accepted under strict 1% FDR cut offs with Scaffold (version Scaffold_4.10.0, Proteome Software Inc., Portland, OR) used to validate MS/MS based peptide and protein identifications. Peptide identifications were accepted if they could be established at greater than 91.0 % probability to achieve an FDR less than 1.0 % by the Scaffold Local FDR algorithm. Protein identifications were accepted if they could be established at greater than 99.0 % probability to achieve an FDR less than 1.0 % and contained at least 2 identified peptides. Protein probabilities were assigned by the Protein Prophet algorithm [1]. Proteins that contained similar peptides and could not be differentiated based on MS/MS analysis alone were grouped to satisfy the principles of parsimony.

1. A. I. Nesvizhskii, A. Keller, E. Kolker and R. Aebersold, *Anal. Chem.*, 2003, **75**, 4646-4658.

Supplementary Methodology – S5

Rheological analysis of cultivation media

Rheological analyses were carried out on cultivation media samples with 15 g L^{-1} NBSK, LP-STEX, and LP-ALKOX, respectively, using an Anton Paar MCR702 rheometer (Anton Paar GmbH, Graz, Austria) thermostatted at 28°C (cultivation conditions). Flow curve measurement were performed in triplicates with parallel plates (d=50 mm). Ostwald-de Waele power law models (Equation S1) were fitted to the flow curves with Matlab 2018b (Mathworks, Natick, MA, USA), using the statistics and machine learning toolbox.

Equation S1. $$\eta = K\dot{\gamma}^{n-1}$$

In Equation S1, η is the apparent viscosity (Pa s), K the consistency index (Pa s^n), $\dot{\gamma}$ the shear rate (s^{-1}), and n the power law index (-). The fitted models were used to describe the linear range of the flow curves. The consistency index describes the overall range of viscosities across the modelled part of the flow curve and equals the apparent viscosity at a shear rate of 1 s^{-1}, which is used in the manuscript as base for comparison of the substrates. The power law index ($0<n<1$) provides a measure of the shear-thinning property of the dispersion. Results are shown in Supplementary Information Figure S2

Supplementary Table – S1

Table S1. Chemical composition of the softwood raw material and prepared substrates. The chemical compositions were determined per National Renewable Energy Laboratory standard methods. Data represents the arithmetic mean ($\bar{x}$) and standard deviation (s) from triplicate analyses expressed as % of dry mass. NBSK: Northern bleached Softwood Kraft pulp, LP-STEX: dilute-acid-catalyzed steam pretreated Lodgepole pine, LP-ALKOX: mild alkali-oxygen pretreated Lodgepole pine, and LP-RM: Lodgepole pine raw material.

	**NBSK**<br>$\bar{x}$ (s)	**LP-STEX**<br>$\bar{x}$ (s)	**LP-ALKOX**<br>$\bar{x}$ (s)	**LP-RM**<br>$\bar{x}$ (s)
Carbohydrates	93.8 (0.5)	52.2 (0.8)	63.9 (0.5)	61.5 (0,6)
Glucan	77.3 (0.3)	51.6 (0.7)	49.7 (0.3)	41.3 (0.2)
Mannan	7.0 (0.2)	0.2 (0.0)	6.6 (0.1)	10.0 (0.5)
Galactan	0.6 (0.0)	0.1 (0.0)	1.4 (0.0)	2.9 (0.0)
Xylan	8.4 (0.1)	0.3 (0.0)	5.2 (0.1)	5.7 (0.1)
Arabinan	0.6 (0.0)	0.1 (0.0	1.0 (0.0)	1.5 (0.0)
Lignin	4.6 (0.0)	46.9 (0.1)	34.6 (0.2)	32.0 (0.3)
Acid-insoluble	0.1 (0.0)	44.4 (0.2)	30.5 (0.0)	28.8 (0.3)
Acid-soluble	4.6 (0.0)	2.6 (0.1)	4.1 (0.1)	3.2 (0.0)
Ash	0.1 (0.0)	0.2 (0.1)	1.0 (0.1)	0.1 (0.0)
Total	98.6 (0.6)	99.4 (0.8)	99.5 (0.4)	93.6 (0.6)

Supplementary Table – S2

Table S2. Substrate morphology and surface properties. Data represents the arithmetic mean ($\bar{x}$) and standard deviation (*s*) from *n* samples.

	n	**NBSK**	**LP-STEX**	**LP-ALKOX**
Substrate morphology				
Fines[a] [%]	3	28.9 (0.5)	80.3 (0.3)	40.0 (0.3)
Mean length [µm]	3×10000	941 (4)	138 (8)	430 (3)
Mean width [µm]	3×10000	46 (9)	44 (11)	47 (10)
Substrate surface properties				
Simons' stain[b] [mg g^{-1}]	4	66 (1.6)	45 (2.7)	67 (0.4)
Total acid groups [µmol g^{-1}]	3	40 (1.5)	23 (1.4)	79 (1.3)
Water retention value[c] [g g^{-1}]	3	1.36 (0.02)	1.13 (0.02)	1.97 (0.01)

[a] Mean length <200 µm

[b] Adsorbed dye, based on substrate dry weight

[c] Retained water, based on substrate dry weight

Supplementary Figures – S1

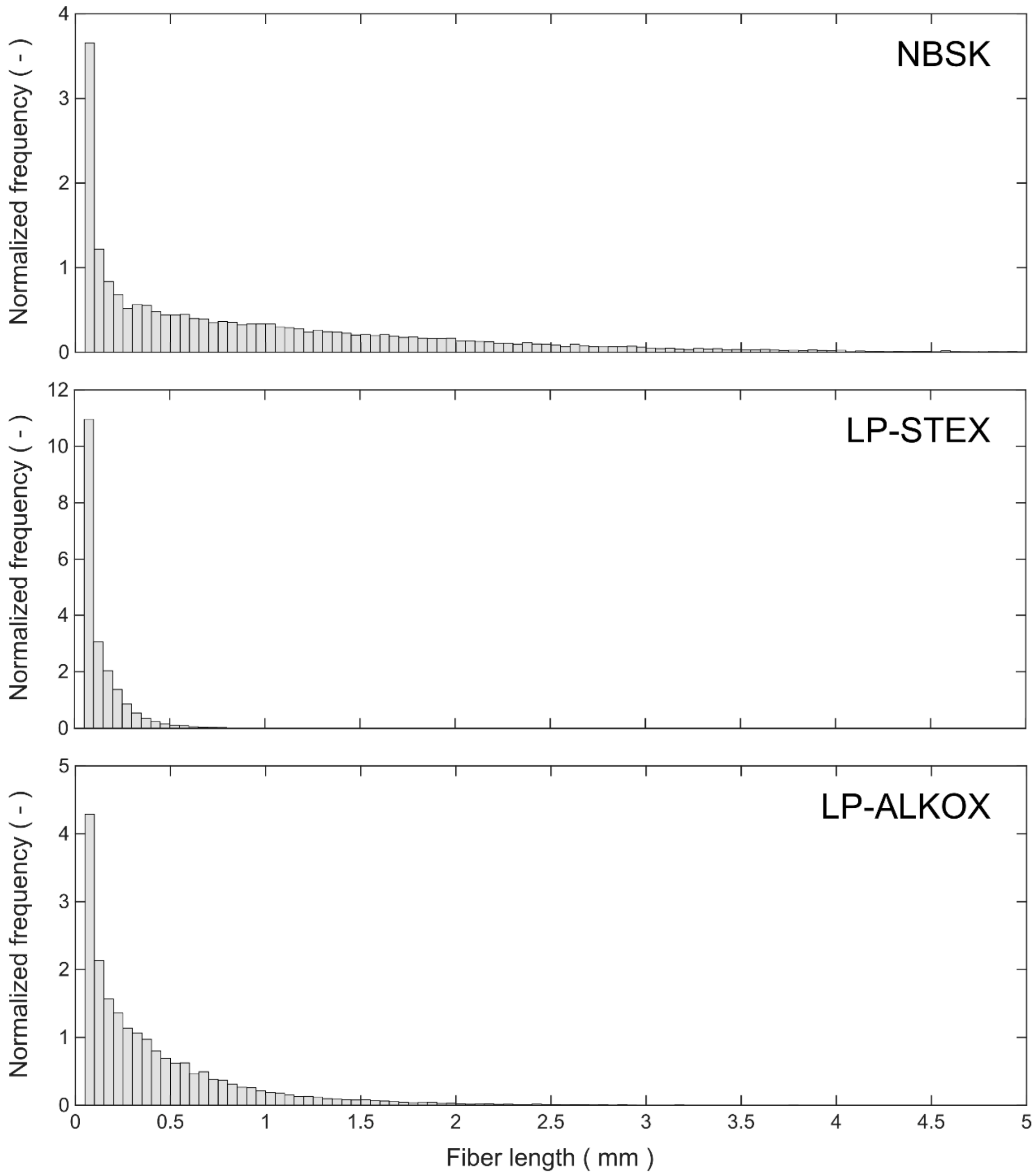

Figure S1. Normalized fiber length distributions (probability density function estimates) of the substrates. Data represents 3×10 000 fibers and was measured with a Fiber Quality Analyzer.

Supplementary Figures – S2

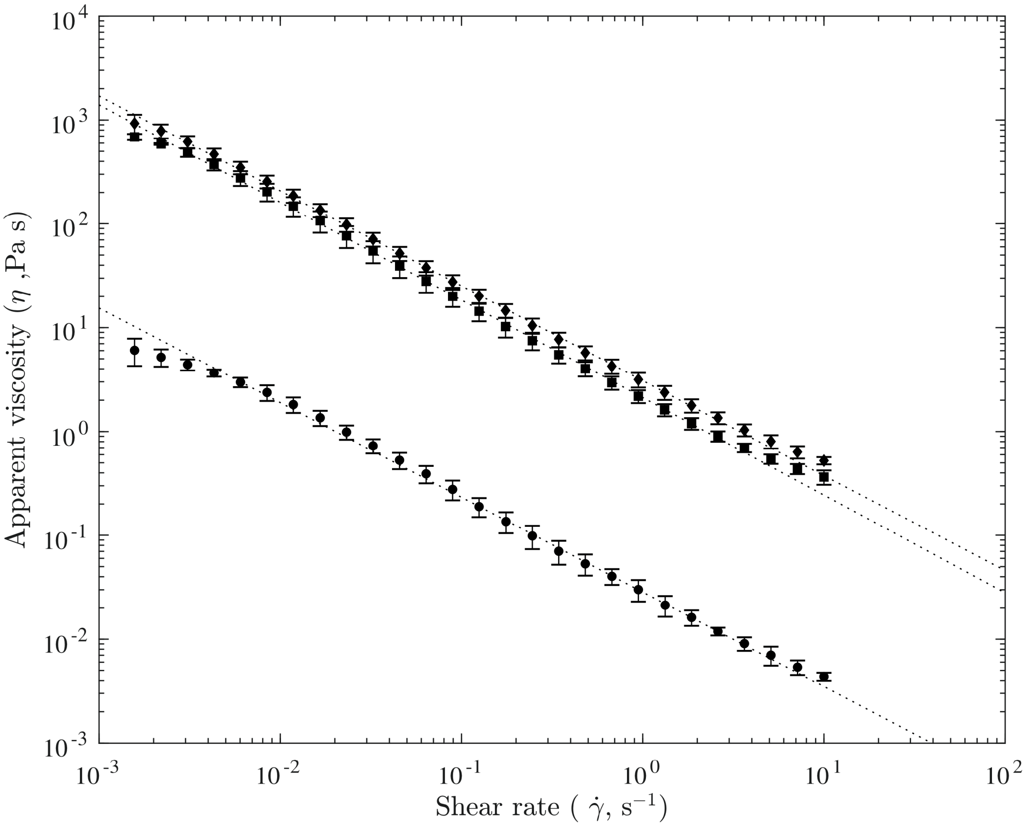

	NBSK	LP-ALKOX	LP-STEX
Consistency index (*K*, Pa s^n)	3.08	2.12	0.03
Power law index (*n*, -)	0.086	0.060	0.088
Data range (-, s^{-1})		0.0031–5.1000	
R^2	0.9941	0.9911	0.9903

Figure S2. Flow curves for 15 g L^{-1} dispersions of NBSK (♦), LP-ALKOX (■), and LP-STEX (●) with fitted Ostwald-de Waele power law models for the linear range and regression statistics. Data in the graph represents the arithmetic mean of triplicates and error bars represent the standard deviation. Data were acquired per Supplementary Method S5.

Supplementary Figures – S3

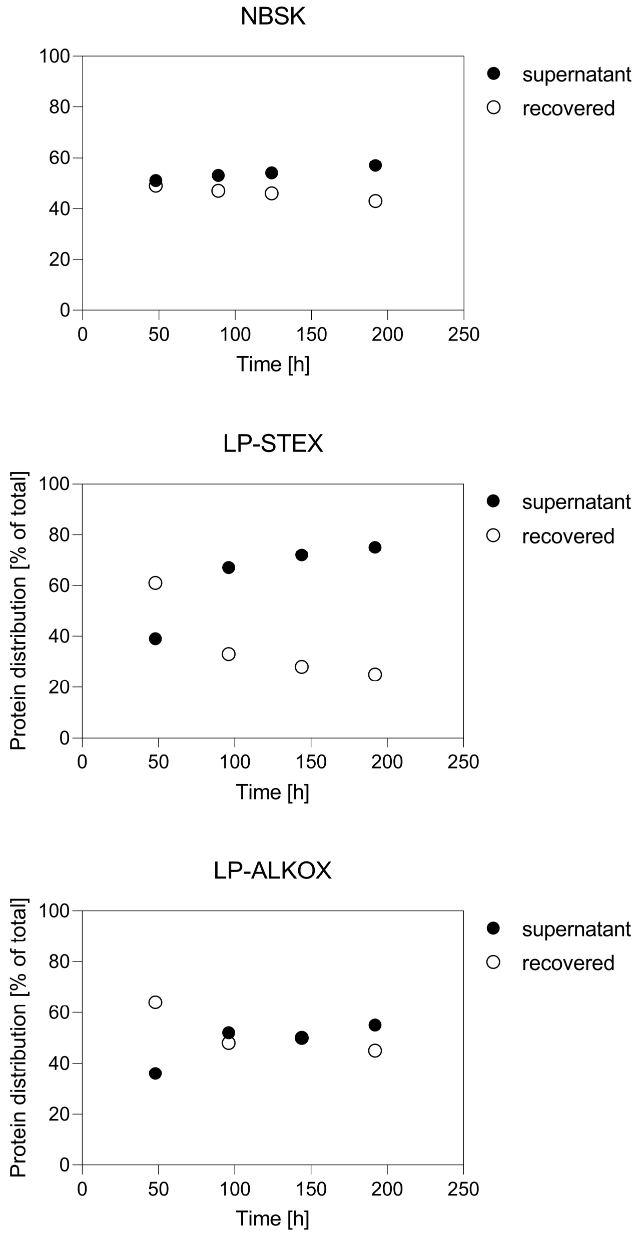

Figure S4. The temporal distributions of total proteins between the supernatant and the fraction desorbed from the insoluble substrate. Cultivation of *T. reesei* QM6a was performed on the three softwood substrates, as indicated. Desorption was performed with Tween 80, as described in the methods section. Depicted are the arithmetic means of ≥2 technical replicates.
